# Spore-FP1 tuberculosis mucosal vaccine candidate is highly protective in guinea pigs but fails to improve on BCG-conferred protection in non-human primates

**DOI:** 10.3389/fimmu.2023.1246826

**Published:** 2023-10-10

**Authors:** Andrew D. White, Andy C. Tran, Laura Sibley, Charlotte Sarfas, Alexandra L. Morrison, Steve Lawrence, Mike Dennis, Simon Clark, Sirine Zadi, Faye Lanni, Emma Rayner, Alastair Copland, Peter Hart, Gil Reynolds Diogo, Matthew J. Paul, Miyoung Kim, Fergus Gleeson, Francisco J. Salguero, Mahavir Singh, Matthias Stehr, Simon M. Cutting, Juan I. Basile, Martin E. Rottenberg, Ann Williams, Sally A. Sharpe, Rajko Reljic

**Affiliations:** ^1^ United Kingdom Health Security Agency (UKHSA), Porton Down, Salisbury, United Kingdom; ^2^ Institute for Infection and Immunity, St George’s University of London, London, United Kingdom; ^3^ Department of Oncology, The Churchill Hospital, Oxford, United Kingdom; ^4^ Lionex GmbH, Braunschweig, Germany; ^5^ School of Biological Sciences, Royal Holloway University of London, Surrey, United Kingdom; ^6^ Sporegen Ltd , London Bioscience Innovation Centre, London, United Kingdom; ^7^ Department of Microbiology, Tumour and Cell Biology and Centre for Tuberculosis Research, Karolinska Institute, Stockholm, Sweden

**Keywords:** tuberculosis, vaccine, lungs, non-human primates, aerosol vaccine

## Abstract

Tuberculosis remains a major health threat globally and a more effective vaccine than the current Bacillus Calmette Guerin (BCG) is required, either to replace or boost it. The Spore-FP1 mucosal vaccine candidate is based on the fusion protein of Ag85B-Acr-HBHA/heparin-binding domain, adsorbed on the surface of inactivated *Bacillus subtilis* spores. The candidate conferred significant protection against *Mycobacterium. tuberculosis* challenge in naïve guinea pigs and markedly improved protection in the lungs and spleens of animals primed with BCG. We then immunized rhesus macaques with BCG intradermally, and subsequently boosted with one intradermal and one aerosol dose of Spore-FP1, prior to challenge with low dose aerosolized *M. tuberculosis* Erdman strain. Following vaccination, animals did not show any adverse reactions and displayed higher antigen specific cellular and antibody immune responses compared to BCG alone but this did not translate into significant improvement in disease pathology or bacterial burden in the organs.

## Introduction

Tuberculosis (TB) remains a global health threat despite the availability of the BCG vaccine, which has been in use for over 70 years. While exerting a good level of efficacy against primary/disseminated TB disease in infants and young children, the BCG vaccine is only partially effective against the pulmonary form of the disease in adults, which is the major source of transmission and disease mortality globally. For these reasons, a consensus view prevails that BCG needs to be either replaced by a more efficacious vaccine or alternatively, boosted with a new vaccine to increase the overall level of protection in the hosts. The latter is probably more realistic, considering that the BCG vaccine is still widely in use, and is given to all infants in TB endemic countries, with no likelihood of discontinuation in the foreseeable future. Therefore, improving BCG efficacy, or complementing BCG with a new boost vaccine would be highly desirable.

Recent studies performed in non-human primates (NHP) suggest that BCG protection could be significantly improved by altering the mode of its delivery (normally intradermal), either by intravenous (1, 2) or the respiratory route (3, 4), though the aerosol route was not superior to the intradermal route in other studies (1). However, it is very likely that a new vaccine will be required to significantly improve control and eventually eradicate TB worldwide. Potential replacement vaccines include MTBVAC (5) and VPM1002 (6) while subunit vaccines (either vectored or protein based) are deemed more suitable as a boost to BCG. Over the past twenty years, many subunit vaccine candidates have been proposed and tested in preclinical and clinical studies, but only two have completed human efficacy trials, with the MVA85A vaccine failing to add to BCG protection in infants (7), and the M72/ASO1 post-exposure vaccine conferring 54% protection against active disease in adults (8). Currently, a number of other vaccine candidates are at different stages of clinical trials (9), while many more are at the preclinical stage of testing and development. Some of the subunit vaccine approaches (clinical and preclinical candidates), among others, include ChadOx1.85A+MVA85A (10), H4:IC31 (11) and ID93/GLA-SE (12), while the therapeutic RUTI vaccine is neither subunit nor whole live cell, but rather based on purified non-toxic fragments from *Mycobacterium tuberculosis* (*Mtb*) (13).

Ordinarily, a new TB vaccine candidate should be protective in one or more of the available animal models of infection (most commonly, mice, Guinea pigs and NHP), before moving to costly and lengthy human clinical trials. However, the capacity of the animal models to replicate human infection and disease, and more importantly, to predict vaccine efficacy in humans remains to be validated. This is further compounded by the absence of reliable correlates of protection. Therefore, testing a vaccine in multiple animal models can provide additional confidence that potential improved efficacy will translate to human trials.

With TB being a respiratory disease, mucosal (inhaled) vaccine delivery might have a better chance of inducing a protective immune response than the systemic route, by virtue of inducing a compartmentalized mucosal immune cell memory, that in turn could provide a rapid response to incipient infection. With that in mind, we previously tested a vaccine formulation based on antigen delivery on the surface of inactivated *Bacillus subtilis* spores via the intranasal route in mice (14–16). Termed Spore-FP1, this formulation, includes a fusion protein made up of three antigens, Ag85B, Acr/HspX and HBHA (Fusion protein 1; FP1), the bacterial spores and the adjuvant PolyI:C. When administered intranasally as a boost to parenteral BCG, this vaccine candidate was able to reduce pulmonary and splenic bacterial infection in mice challenged with low dose aerosolized H37Rv strain of *M. tuberculosis,* (14). Ag85B and Acr antigens were selected as representatives of active and latent infection, and the N-terminal domain of HBHA (heparin-binding hemagglutinin adhesion), was included to facilitate interaction with the pulmonary epithelium.

The adjuvant activity in Spore-FP1 is conferred by both the spores themselves and the PolyI:C adjuvant. Thus, spores were shown to activate transcription factors downstream of Toll-like receptor signaling, including AP-1 (c-JUN), NF-kB and IRF-3, leading to activation of dendritic cells (DC) and macrophages, as measured by detection of enhanced expression of CD80, CD86, MHC-I, CCR7 and PDL-1 (14). PolyI:C, a well-known inducer of TLR3 signaling and cell mediated immunity, has been shown to effectively compartmentalize T cell responses to the lungs of non-human primates (NHP) (17). Furthermore, the inclusion of the heparin-binding domain of HBHA (18) within the FP1 fusion protein, is intended to facilitate interaction of FP1-coated spores with alveolar lung epithelial cells, potentially leading to vaccine translocation across the epithelium in much the same manner the pathogen itself does, gaining access to mucosal immune cells in the lamina propria.

Having previously demonstrated Spore-FP1 efficacy as a mucosal vaccine in the mouse model of *Mtb* infection (14), we set out to test it in Guinea pigs and NHP. We also used human IgG receptor transgenic mice (19) to specifically address the potentially protective role of Spore-FP1 induced antibodies. Due to its potential as a mucosal vaccine candidate, we therefore wanted to include the inhaled delivery in these animal models, with aerosolized delivery in NHP being the main target. As a protein-based subunit vaccine, little prior information was available for aerosolized delivery in the context of TB vaccines, so an important technological challenge was to determine the parameters affecting the aerosolization process and vaccine recovery, as well as dose control in the NHP. In this report, we present evidence for the immunogenicity and protective potential of Spore-FP1 in Guinea pigs and NHP but also draw attention to certain limitations of the aerosolized delivery of non-live vaccine candidates in NHP that need to be overcome to further advance this vaccine approach.

## Materials and methods

### Spore-FP1 vaccine

Spore-FP1 vaccine consists of heat-inactivated *Bacillus subtilis* spores (strain HU58), the fusion protein FP-1 (Ag85B-Acr-HBHA) and PolyI:C (Sigma-Aldrich, Dorset, UK) as adjuvant. It should be noted that only the short (6 kDa) heparin-binding domain of HBHA was included, to facilitate the epithelial adhesion/translocation (18), and consequently, immune responses to HBHA have not been monitored. Further characterization of this vaccine can be found in our previous report (14). For aerosolization studies in NHP, the vaccine formulation was nebulized using an Omron MicroAIR NE-U22-E device, and the spore-immobilized protein in the condensate verified by analytical methods. Optimal parameters for nebulized volume, delivery time and percent recovery to deliver the required dose were established *in vitro*.

### Animals, approvals, and experimental protocols

#### Mice

3–9-month-old male and female human CD64 transgenic mice (BALB/c background) were used in the study (19). All animals were used with approval from St George’s University of London Ethics Committee under an approved UK Home Office animal project license and used in accordance with the UK Animals (Scientific Procedures) Act 1986. Groups consisted of at least 6 animals, following power calculations based on the minimal anticipated magnitude of the effect of 0.5 log difference, 90% statistical power and an average intra-group variability from previous experiments of 1 log. Mice were housed at the St George’s University of London Biological Research Facility and transferred to a CL3 facility for challenge with aerosolized *Mtb*. FP1-specific antibodies from serum of Spore-FP1 vaccinated NHPs were purified using an FP1-coupled Affigel-15 (BioRad, UK) chromatography column.

Mice were divided into 3 groups (PBS n=7; IFN-γ + naïve IgG, n=6; IFN-γ + FP1 Ig n=6). Animals in treatment groups received an intranasal treatment 2 hours before infection, consisting of 30µg antibody combined with 1µg per animal of IFN-γ (Peprotech, USA). Mice were then infected by aerosolized *Mtb* H37Rv (UKHSA NCTC 7416), using the Biaera Technologies nose-only system, receiving approximately 230 bacilli per animal. Animals were given two further treatments 2 and 7 days after H37Rv challenge. All animals were then culled by cervical dislocation 7 days after the final treatment and lungs were excised for CFU enumeration. Lungs were homogenized in PBS 0.1% w/v Triton X-100 in a Precellys homogenizer (Bertin Instruments, France) and plated in duplicate on 7H11 agar plates to enumerate CFU.

#### Guinea pigs

Studies were conducted according to the United Kingdom (UK) Home Office, or Sweden Legislation for animal experimentation and approved by a local ethics committee at the UK Health Security Agency (UKHSA) and the Stockholm’s Region Animal Research Ethic Committee no. 210-14. Dunkin Hartley strain guinea pigs weighing between 200-300g and free from pathogen-specific infection were purchased from an approved supplier (Envigo, UK, and Lidköping Farms, Sweden) and used in this study. Groups consisted of seven or eight animals per group based on statistical power calculations (Minitab, version 16) performed to determine group sizes using previous data (SD, approximately 0.5) to reliably detect a difference of 1.0 log_10_ in the group mean number of CFU/ml. Animals were randomly assigned to vaccine groups and identified using subcutaneously implanted microchips (Plexx) to enable blinding of the analyses wherever possible. Throughout the study, animals were housed in the Biological Investigations Group facility at UKHSA, and the animal facility at Karolinska Institutet (KI).

BCG and Spore-FP1 vaccines were prepared on the day of vaccination. Eight (UKHSA, homologous vaccination) or seven (Karolinska, BCG prime-Spore-FP1 boost) animals were vaccinated once subcutaneously on the nape with 100µl of Spore-FP1 vaccine (containing 20 µg FP1, 1x10^9^ spores and 20 µg polyI:C), or 5×10^4^ CFU in 250 µl of BCG Danish 1331 (UKHSA), or 1x10^5^ CFU BCG Pasteur (KI). Some animals remained unvaccinated as a negative control. Three weeks following Spore-FP1 prime-vaccination, or 10 weeks following BCG prime-vaccination, the Spore-FP1 and BCG+Spore-FP1 vaccine group were boosted twice, three weeks apart with Spore-FP1 (100µl) delivered by the intranasal route. All animals were rested for a further six weeks prior to challenge with *Mtb*. Guinea pigs were infected with a low aerosol dose (10–20 CFU retained dose in the lung) of *Mtb* H37Rv NCTC 7416 (UKHSA) or Harlingen strain (KI). At UKHSA, aerosol challenge was performed using a fully contained Henderson apparatus as previously described (20) in conjunction with the AeroMP (Biaera) control unit (21). Fine particle aerosols of *Mtb*, with a mean diameter of 2 µm (diameter range 0.5–7 µm) (20) were generated using a 3-jet Collison nebulizer and delivered directly to the animal snout. The Henderson apparatus allows controlled delivery of aerosols to the animals and the reproducibility of the system and relationship between inhaled CFU and the concentration of organisms in the nebulizer has been described (20). At KI, custom-made individual restrainers coupled to a nose only aerosol exposure unit (20 min) (In-tox Products, Moriarty, NM, US) were used for infection of GPs at KI. The aerosol was generated from a water suspension containing 5x10^6^ CFU/ml of *Mtb, and* animals exposed to aerosol for 20 min. Animals were subsequently transferred to housing within ACDP containment level 3 facilities.

#### Non-human primates

A total of 36 macaques were used in a series of three studies designed to assess safety, vaccine optimization, immunogenicity, and efficacy. All studies used male rhesus macaques (*Macaca mulatta*) of Indian origin, sourced from an established, closed UK breeding colony. Macaques assigned to the vaccine safety and optimization studies: macaque study 1 (n=8) and macaque study 2 (n=9) were aged between 4.2 and 4.5 years, while macaques assigned to the vaccine immunogenicity and efficacy against aerosol *Mtb* challenge study (macaque study 3, n=18) were between 3.7 and 3.9 years at the time of enrolment. Absence of previous exposure to mycobacterial antigens was confirmed by screening with an IFN-γ ELISPOT assay (MabTech, Nacka. Sweden), using tuberculin-PPD (SSI, Copenhagen, Denmark), and pooled 15-mer peptides of ESAT6 and CFP10 (Peptide Protein Research LTD, Fareham, U.K.). Male PPD non-responders within the ages indicated were then randomized between experimental groups.

Compatible social groups, consisting of males only, were housed in accordance with the Home Office (UK) Code of Practice for the Housing and Care of Animals Bred, Supplied or Used for Scientific Purposes (2014), and the National Committee for Refinement, Reduction and Replacement (NC3Rs) Guidelines on Primate Accommodation, Care and Use, August 2006 (NC3Rs, 2006). Cages were approximately 2.5m high by 4m long by 2m deep, constructed with high level observation balconies and with a floor of deep litter to allow foraging. Additional environmental enrichment was afforded by the provision of toys, swings, feeding puzzles and DVDs for visual stimulation. In addition to standard old-world primate pellets, diet was further supplemented with a selection of fresh vegetables and fruit. For each procedure, sedation was applied by intramuscular injection with ketamine hydrochloride (10 mg/kg) (Ketaset, Fort Dodge Animal Health Ltd, Southampton, UK). None of the animals had been used previously for experimental procedures and each socially compatible group was randomly assigned to a particular study treatment. The study design and all procedures were approved by the UK Health Security Agency, Porton Down Animal Welfare and Ethical Review Body, and authorized under an appropriate UK Home Office project license.

Animals were sedated at regular intervals for blood sample collection and to measure body weight and temperature, red blood cell (RBC) hemoglobin levels and erythrocyte sedimentation rate (ESR). RBC hemoglobin levels were monitored as an indication of anemia, measured using a HaemaCue haemoglobinometer (Haemacue Ltd, Dronfield, UK). ESR was monitored using the Sediplast system (Guest Medical, Edenbridge, UK) as a general measure of *Mtb* induced inflammation. Bronchoalveolar lavage (BAL) fluid was collected at selected timepoints following Spore-FP1 vaccination during the optimization and vaccine immunogenicity studies, as described previously (22). Animal behavior was observed throughout the studies for contra-indicators and the time of necropsy, if prior to the end of the planned study period, was determined by experienced primatology staff based on a combination of the following adverse indicators: depression or withdrawn behavior abnormal respiration (dyspnoea), loss of 20% of peak post-challenge weight, ESR levels elevated above normal (>20 mm), hemoglobin level below normal limits (< 100g/dL), increased temperature (>41°C) and abnormal thoracic radiograph.

BCG vaccinations were delivered to sedated macaques in the upper left arm as a 100 μl intradermal (ID) injection. BCG Danish strain 1331 (AJ Vaccines, Copenhagen, Denmark) was used for macaque vaccine safety and immunogenicity study one, and for the BCG+Spore-FP1 vaccine efficacy experiment (study 3), whereas BCG Bulgaria strain SL222 (Intervax, Markham, Ontario, Canada) was used for the second macaque vaccine immunogenicity study (macaque study 2). The titer of BCG vaccine preparation was confirmed to be within the range specified by the manufacturer by bacterial culture on Middlebrook 7H11 selective agar containing oleic acid, bovine albumin, dextrose, and catalase (OADC) (Biomerieux, Basingstoke, UK), for enumeration of viable colony forming units (CFU).

Low dose spore-FP1 vaccinations were prepared by mixing 2x10^9^ heat inactivated *Bacillus subtilis* spores (SGUL, London, UK) with 60 µg of the mycobacterial antigen fusion protein FP1 (Lionex, Braunschweig, Germany) and 40 µg of the synthetic double-stranded RNA (dsRNA) adjuvant compound PolyI:C (Sigma-Aldrich, Dorset, UK) all diluted in sterile saline solution. Two ml of vaccine preparation was delivered by aerosol using an Omron MicroAir mesh nebulizer (Omron Healthcare UK Ltd, Milton Keynes, UK) and a modified pediatric anaesthesia mask, or as a 182 μl volume delivered by ID injection in the upper right arm. High dose Spore-FP1 vaccinations were prepared by mixing 1x10^10^ *Bacillus subtilis* spores with 300 µg of FP1 fusion protein and 40 µg PolyI:C in sterile saline solution and delivered by aerosol using an Omron MicroAir mesh nebulizer.

#### Aerosol *M. tuberculosis* challenge of macaques

Animals assigned to the BCG + Spore-FP1 vaccine efficacy experiment (macaque study 3), were challenged by exposure to aerosols of *Mtb* as previously described (23), twenty-one weeks after BCG vaccination. Mono-dispersed bacteria in particles were generated using a three-jet Collison nebulizer (BGI) and, in conjunction with a modified Henderson apparatus (24), delivered to the nares of each sedated primate via a modified veterinary anesthetic mask. Challenge was performed on sedated animals placed within a ‘head-out’, plethysmography chamber (Buxco, Wilmington, North Carolina, USA) to enable the aerosol to be delivered simultaneously with the measurement of respiration rate and respired volume. The calculations to derive the presented dose (the number of organisms that the animals inhale) and the retained dose (the number of organisms assumed to be retained in the lung) have been described previously (25). The presented aerosol challenge dose applied was quantified as an average of 16 CFU (range 12–20 CFU) *Mtb* Erdman.

Animals were monitored daily, and blood samples collected at regular intervals throughout the study for evaluation of immune responses. A range of clinical and behavioral parameters were assessed and where necessary used to determine whether the disease related changes met end-point criteria for euthanasia. At necropsy, TB-induced disease pathology was assessed and scored using a range of macro- and microscopic techniques and viable *Mtb* CFU recovered from extra-thoracic tissues quantified for comparison between experimental groups.

### Computed tomography imaging of NHP

Computed Tomography (CT) scans were performed on sedated animals at 3, 7, 11 and 15 weeks following infectious challenge (16 slice Lightspeed CT scanner, GE Healthcare, Milwaukee, WI, USA), as previously described (23). Niopam 300 (2ml/kg i.v., Bracco, Milan, Itay) was used as a contrast agent. An expert medical consultant radiologist with expertise in respiratory diseases, including in non-human primates assessed the scans blinded to the animals’ treatment and clinical status. The number and lobar distribution of pulmonary lesions and other pathological abnormalities including nodular conglomeration, cavitation, consolidation (associated with alveolar pneumonia), lobular collapse, and the ‘tree-in-bud’ pattern associated with bronchocentric pneumonia, were recorded. Upper airways were assessed for bronchocele and wall thickening. Other body tissues were scored for enlargement, necrosis, cavitation and extrapulmonary foci of infection: lung associated lymph nodes, liver, kidneys, and spleen. Findings were scored, and composite scores for tuberculosis disease burden (a relative score comprising the following factors; number of lesions, presence and extent of physiological changes (26) pneumonia (sum of consolidation & tree-in-bud morphology scores) and total CT score (the sum of scores attributed to each tissue) were calculated.

### IFN-γ ELISPOT

Peripheral blood mononuclear cells (PBMC) were isolated using standard methods from heparin anti-coagulated blood collected from macaques. An IFN-γ ELISpot assay was used to quantify the number of mycobacteria-specific IFN-γ-producing T-cells in PBMCs using a human/simian IFN-γ kit (MabTech, Nacka. Sweden), as described previously (2). In brief, 2 × 10^5^ PBMCs were cultured with 10 μg/ml PPD (AJ Vaccines, Denmark), 20 μg/ml Ag85B, 20 μg/ml FP-1 fusion protein (both from Lionex, Braunschweig, Germany) or pools of overlapping 15-mer peptides spanning CFP10 or ESAT6 (Peptide Protein Research Ltd, Wickham, UK) in duplicate, or without antigen, in quadruplicate, and incubated for 18 h. Phorbol 12-myristate (Sigma-Aldrich Dorset, UK) (100 ng/ml) and ionomycin (CN Biosciences, Nottingham, UK) (1 μg/ml) were used as a positive control. After culture, spots were developed according to the manufacturer’s instructions. Plates were scanned, and spots enumerated using a CTL Immunospot S6 reader and software. Determinations from replicate tests were averaged, and the data were analyzed by subtracting the mean number of spots in the medium-only control wells from the mean counts of spots in wells with antigen, or peptide pools, to derive an antigen-specific spot count. This value was multiplied by a factor of five and reported as IFN-γ SFU frequency per million PBMCs. Antigen-specific IFN-γ SFU profiles were plotted using Graphpad v8.0.1 (Graphpad Inc, USA).

### ICS assays

#### Polyfunctional intracellular cytokine staining and antigen-specific memory T-cell assay

Intracellular cytokine staining (ICS) was performed using 1 x 10^6^ PBMC in medium (R10) consisting of RPMI 1640 supplemented with L-glutamine (2 mM), penicillin (50 U/ml) streptomycin (50 μg/ml) (all from Sigma Aldrich, Gillingham, UK) and 10% heat-inactivated fetal bovine serum (Labtech Ltd, Uckfield, UK). These cells were stimulated with a 10 μg/ml solution of CD28 and CD49d co-stimulatory antibodies (both from BD Biosciences, Oxford, UK) and either 10 μg/ml PPD (AJ Vaccines, Denmark), 20 μg/ml Ag85B (Lionex, Braunschweig, Germany) or 5 μg/ml staphylococcal enterotoxin B (SEB) (Sigma Aldrich, Gillingham, UK), or R10 medium alone as negative control, for a total of six hours at 37°C, in a 5% CO_2_ supplemented incubator. Following the initial two hours of incubation, the protein transport inhibitor Brefeldin-A (Sigma Aldrich, Gillingham, UK) was added to the incubation mixture at a final concentration of 10 μg/ml. Following incubation, cells were washed with FACS buffer consisting of PBS + 1% FCS and incubated for 30 minutes at room temperature with optimal dilutions of the amine-reactive Live/Dead Fixable Red viability cell stain (Life Technologies, Renfrew, UK) and the antibodies CD4 PerCP-Cy5.5, CD8 APC-Fire 750, CD95 Pe-Cy7 (all from BD Biosciences, Oxford, UK), CD28 BV-421, CD16 BV-786, CD56 BV-605 (Biolegend, London, UK), CCR7-PE (eBioscience, Hatfield, UK), CD14-ECD and CD20-ECD (both from Beckman Coutler, High Wycombe, UK). Following surface marker staining, the cells were washed and then permeabilized by incubation at RT for 15 minutes with Fix/Perm reagent (BD Biosciences, Oxford, United Kingdom). Further cell washes were applied using Permwash buffer (BD Biosciences, Oxford, United Kingdom), before staining for intracellular antigens by incubation at RT for 30 minutes with the antibodies CD3-AF700, IFN-γ-FITC, TNF-α-BUV395 (all from BD Biosciences, Oxford, United Kingdom), IL-2-APC (Miltenyi Biotech Ltd, Bisley, UK), IL-17-BV711 (Biolegend, London, UK). BD Compbeads (BD Biosciences, Oxford, UK) were labelled with the above fluorochromes for use as compensation controls. Following antibody labelling, cells and beads were washed by centrifugation and fixed in 4% paraformaldehyde solution (Sigma Aldrich, Gillingham, UK) prior to flow cytometric acquisition.

#### Flow cytometric acquisition and analysis

Cells were analyzed using a five laser LSRII Fortessa instrument (BD Biosciences, Oxford, UK) and data were analyzed using FlowJo (version 9.7.6, Treestar, Ashland, US). Cytokine-producing T-cells were identified using a forward scatter-height (FSC-H) versus side scatter-area (SSC-A) dot plot to identify the lymphocyte population, to which appropriate gating strategies were applied to exclude doublet events, non-viable cells, monocytes (CD14^+^) and B cells (CD20^+^). For ICS analysis, sequential gating through CD3^+^, followed by CD4^+^ or CD8^+^ gates were used to identify T-cell populations, or CD3^−^, CD8^+^/CD56^+^ and/or CD16^+^ NK cells populations, before individual cytokine gates to identify IFN-γ, IL-2, TNF-α and IL-17 producing populations. Antigen-specific T-cell memory profiles were identified by applying a summed CD4 or CD8 cytokine Boolean combination, followed by gating for CD95 surface staining. Differentiation of effector, transitional effector and central memory T-cell populations was established by CD28 and CCR7 expression patterns (**Figure S1**). The software package PESTLE version 1.7 (Mario Roederer, Vaccine Research Centre, NIAID, NIH) was used for background subtraction to obtain antigen-specific polyfunctional ICS and memory T-cell cytokine responses, Graphpad Prism (version 8.0.1) was used to generate graphical representations of flow cytometry data.

#### Necroscopy

Guinea pigs were killed by an overdose with sodium pentobarbital given by the intraperitoneal route. At necropsy, lungs and spleens were removed as described previously (27) and lobes/sections divided between bacteriology and histology analyses. Representative samples of the left middle, left caudal, right middle and right accessory lung lobes, and the spleen, were sampled consistently and placed in 10% neutral buffered formalin (NBF) for histological processing and evaluation.

Necropsies were performed on all macaques in which disease reached levels that met humane endpoint criteria or at the pre-defined study end date 16 to 18 weeks after *Mtb* aerosol challenge. Following anesthesia, clinical data and blood samples were collected. Euthanasia was performed with 140mg/kg IC lethal dose of pentobarbital (Dolethal, Vétoquinol UK Ltd) administered via the intra-cardiac route. Examination of the tissues was performed immediately following the same criteria as described above (CT imaging), using an established necropsy score for visual TB disease pathology (28). The following tissues were sampled for quantitative microbiology: spleen, liver, kidneys, tracheobronchial, inguinal, and axillary lymph nodes. The thoracic ‘pluck’, comprising lung, heart and trachea was fixed with 10% NBF using a syringe and 13CH Nelaton catheter (J.A.K. Marketing, York, UK) for pathology, by inserting the catheter into each bronchus and injecting fixative until the lung expanded to the perceived usual inspiratory dimensions. The trachea was ligated, and the lungs immersed in 10% NBF. Samples of kidneys, liver, spleen, and lymph nodes (sub clavicular, hepatic, inguinal and axillary) were also fixed in 10% NBF for histological processing and evaluation.

### Pathological studies

#### Gross examination of formalin-fixed macaque tissues

Each lung lobe from individual animals were identified and removed from the thoracic ‘pluck’ and sliced consecutively at approximately 5mm thickness using a standardized orientation. The number of discrete lesions and the dimensions of coalesced lesions were recorded. A section of each lobe that contained visible lesions or, if lesions were absent, from a pre-defined site in each lobe, was selected for histopathological examination; lung-associated lymph nodes were also sampled.

#### Histopathological examination

For guinea pigs, tissue representative of each lung and spleen, sampled consistently between animals, was processed routinely (by formaldehyde fixation) and embedded in paraffin wax. Sections (thickness of approximately 5 µm) were stained with hematoxylin and eosin. the severity of lesions was assessed with the pathologist blinded to the treatment groups using a subjective scoring system as described elsewhere (27). Briefly, for the spleen, a subjective scoring system based on number of lesions, was calculated. For the lung, each lobe was assigned a score as follows: 0, normal; 1, very few or small lesions, 0–10% consolidation; 2, few or small lesions, 10–20% consolidation; 3, medium sized lesions, 20–33% consolidation; 4, moderate sized lesions, 33–50% consolidation; 5, 50–80% consolidation, extensive pneumonia, >80% consolidation; for each category, the number of foci of necrosis was also recorded. Scores from each lobe were combined and a mean score from lung lobes and spleen was calculated for each group (27).

For NHP, all samples were processed to paraffin wax blocks using standard procedures, sectioned at ~4 µm, stained with hematoxylin and eosin (H&E) and Ziehl-Neelsen (ZN) stains. Slides were scanned digitally using ‘3D Histech’ slide scanner and proprietary ‘Caseviewer’ software used to capture, store, and annotate digital images. All gross and histopathological examinations were carried out by a qualified veterinary pathologist blinded to the treatment group. The TB-associated lesions were recorded using the scoring system described by Rayner et al. (29). Briefly, six different types of granulomas were identified. Types one (I), two (II) and three (III) were considered as “unorganized” lesions, while types four (IV), five (V) and six (VI) were described as “organized” granulomas. Type I comprised small, diffuse foci of macrophages and lymphocytes with scattered neutrophils and eosinophils, lacking clearly defined margins, infiltrating alveolar walls and extending into alveoli. Type II lesions were composed of similar cell types as type I but were larger in size and forming a more defined circumscribed focus of granulomatous inflammation, frequently circular and with variably demarcated borders. Type III were similar to type II but with central. focal necrosis, characterized by nuclear pyknosis and karyorrhexis with the loss of cellular architecture. Type IV comprised circumscribed granulomas consisting primarily of macrophages admixed with neutrophils and other leucocytes, and with a rim of lymphocytes around the periphery. Type V were similar to type IV with a central focus of necrosis containing nuclear pyknosis, karyorrhexis and cellular debris; and type VI were classic, largely well-demarcated, granulomas with central, caseous necrosis and a variable rim of lymphocytes. For each tissue section, the total area of lung parenchyma affected by tuberculous changes was calculated and the total number of granulomas of each type were counted and recorded. Moreover, the total number of AFBs were counted for each granuloma in the ZN-stained sections from the lung and lung-associated lymph nodes.

### Bacteriology

For guinea pig and mouse studies, protection was measured by comparing bacterial load between groups, four (guinea pigs) or two (mice) weeks after challenge. Animals were killed by an overdose with sodium pentobarbital given by the intraperitoneal route (i.p.) (GP) or cervical dislocation (mice). At necropsy, lungs and spleens were removed as described previously (27), and lobes/sections divided between bacteriology and histology analyses. For bacterial load analysis, each tissue was homogenized in 2 ml sterile PBS (1x). Each tissue homogenate sample was serially diluted in sterile PBS (1x) and 100 µl of each dilution was plated in duplicate onto Middlebrook 7H11 OADC selective agar. Plates were incubated at ~37°C for up to 4 weeks. Following incubation, colonies were enumerated (as CFU), and the concentration of bacilli in each sample homogenate was calculated. Bacterial load data were expressed as log_10_ CFU/ml.

For NHP studies, spleen, kidneys, liver and lung-associated lymph nodes were sampled for the presence of viable *Mtb* at necropsy, as described previously (30). Weighed tissue samples were homogenized in 2 ml of sterile water, then either serially diluted in sterile water prior to being plated or plated directly onto Middlebrook 7H11 OADC selective agar. Plates were incubated for three weeks at 37 °C and resultant *Mtb* colonies counted. Mean CFU counts from replicate tissue samples were used for graphical presentation.

### Antibody analysis and purification

Vaccine-specific antibody responses were determined by ELISA. 96-well ELISA plates (NUNC, Thermo Fisher Scientific, UK) were coated with 5µg/ml of antigen. Nasal swabs (collected from both anterior nares using sterile cotton buds and stored in 1 ml of PBS + 0.1% BSA supplemented with penicillin-streptomycin) and bronco-alveolar lavage samples were prepared by vortexing in PBS for 1 minute, then centrifuging for 5 min to remove cellular debris. Serum and mucosal samples were loaded at 1:2 – 1:100 starting dilution, and further 3-fold serial dilutions were performed. Antigen-specific NHP antibodies were detected using horseradish peroxidase (HRP) conjugated anti-NHP IgA, IgG or IgM antibodies (Sigma), and developed using SigmaFast OPD (Sigma). Absorbance was measured at 450nm.

NHP antibodies were purified from pooled sera of immunized animals. Samples were applied on an affinity column with immobilized FP1. Bound proteins were eluted with 0.1M glycine pH 2 followed by immediate neutralization by addition of 1M TRIS pH 9 buffer. Elution fractions were then pooled and dialyzed using a cassette (Thermo Scientific, UK) against PBS pH 7.4 overnight and concentrated with a 30kDa centrifugal concentrator (Amicon-Merck, Watford UK). Purified FP1-specific antibodies were characterized by antigen ELISA described above, and SDS-PAGE using 4-12% BIS-TRIS gels under non-reducing and reducing (β-mercaptoethanol) conditions. The gels were stained using Coomassie blue (Expedeon, Cambridge, UK).

### Biacore assays

Binding of NHP antibodies to recombinant human CD64 receptor was investigated by surface plasmon resonance (SPR) using the BIAcore X100 (GE Healthcare, USA) instrument. Protein A (Sigma) was immobilized onto both flow cells of a Biacore carboxymethyldextran (CM5) chip at a target level of 5000 response units (RU). Human or NHP IgG at 20µg/ml was then flowed over the test flow cell to 500RU after which various concentrations of recombinant human CD64 were flowed over the chip at a flow rate of 10µl/min to determine affinity. The biosensor surface was regenerated with 10mM glycine pH 2.0 three times at 10µl/min to elute both IgG and CD64, exposing the Protein A surface for subsequent runs. The data from both cells were used in the analysis of kinetics, with a control flow cell (Protein A only) providing readings of background fluctuations caused by change in buffer or unspecific binding. Data analysis was carried out using BIAevaluation 2.0.1 (GE Healthcare, USA).

### Statistical analyses

For NHP studies, GraphPad Prism version 8.0.1 (GraphPad Software Inc, La Jolla, California, USA) was used for all graphical data representations and statistical analyses, these included: comparison of antigen-specific IFN-γ SFU frequencies measured using the ex vivo ELISpot assay, frequencies of specific cell subsets and multifunctional cell populations measured by flow cytometry, differences in qualitative pathology scores and histopathology granuloma severity scores, quantification of viable *Mtb* in extrapulmonary tissues and comparisons of clinical parameters between vaccination groups, using non-parametric Wilcoxon signed-rank or Mann–Whitney *U* tests. Cochran–Armitage method *X* ^2^ tests were used to compare the frequency of granuloma severity data between the vaccination groups. Disease progression between groups was compared using log-rank survival analysis. Where applicable, all statistical analyses were conducted using two-tailed tests and *P* values are unadjusted for multiple comparisons.

For guinea pig studies, and the passive transfer experiment in mice, normality tests were performed using the Shapiro-Wilk method. Group mean histopathology scores and bacterial load data were compared between groups. Statistical significance of bacteriology and histopathology data were assessed using one-way ANOVA using Tukey’s *post hoc* test.

## Results

### Spore-FP1 is protective in guinea pigs

**Figure 1:**
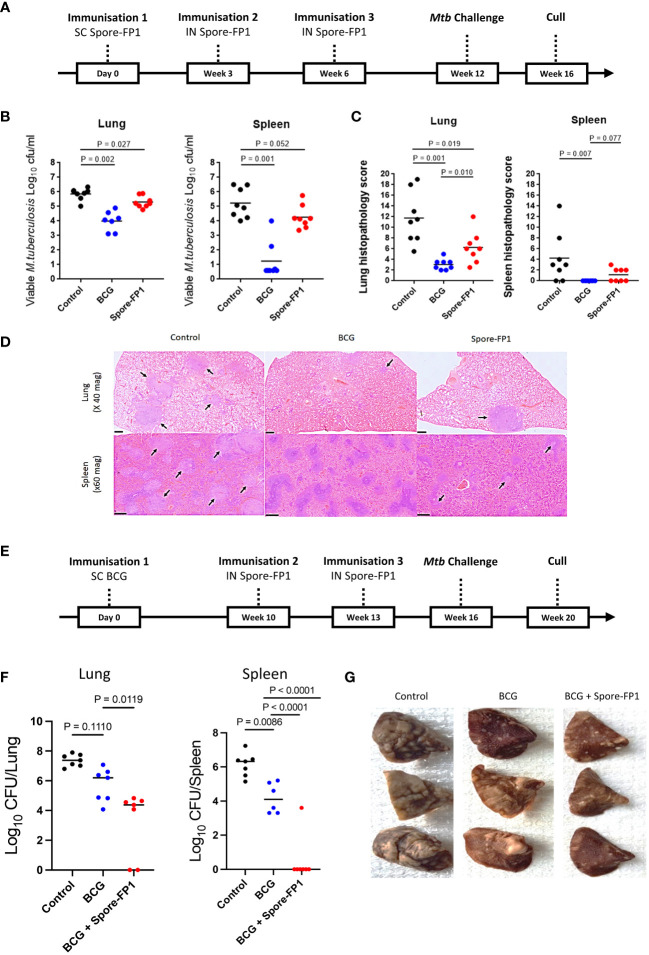
Protection conferred by Spore-FP1 in guinea pigs. **(A)** Schematic representation of the Spore-FP1 homologous immunization regimen and *Mtb* challenge; **(B)** Viable *Mtb* counts in representative lung sections and spleen (shown are the means and individual animal counts, n=8); **(C)** Lung and spleen histopathology scores; **(D)** Representative lung and spleen section stained with hematoxylin and eosin (H&E), with the arrows pointing to granuloma formations; **(E)** Schematic representation of the BCG prime - Spore-FP1 boost heterologous immunization regimen and *Mtb* challenge; **(F)** viable *Mtb* in the lungs and spleen (n=6-7); **(G)** Gross pathology of the lungs. Statistical analysis: One-way ANOVA and Mann-Whitney U test using GraphPad prism version 7; P<0.05 represents significant difference.

Guinea pigs were vaccinated with Spore-FP1 vaccine given on three separate occasions, three weeks apart using a SC/IN/IN route regimen. Animals were rested for six weeks prior to infection by the aerosol route with *Mtb* H37Rv. Four weeks post-infection, animals were culled, and bacterial burden and histopathology were analyzed in lungs and spleen (schedule shown in **Figure 1A**).

Vaccination of guinea pigs with Spore-FP1 gave significant protection compared with the unvaccinated control group when measured by reduction in bacterial burden in the lung (*p*=0.027) and histopathology score (*p*=0.019) in the lung and spleens (**Figures 1B, C**). A similar trend in protection from Spore-FP1 was observed when bacterial burden was measured in the spleen (*p*=0.052). Vaccination with BCG (positive control) gave significant protection in both lung and spleen measured by bacterial load (*p*=0.002 and *p*=0.001) and histopathology score (*p*=0.001 and *p*=0.007), respectively. Photomicrographs (x20 magnification) representing the group mean pathology demonstrate a reduction in numbers of granulomas in the lungs and spleens of Spore FP-1 and BCG vaccinated animals compared to the unvaccinated group (**Figure 1D**).

We next tested Spore-FP1 as a boost to BCG immunization in guinea pigs challenged with *Mtb*, according to schedule shown in **Figure 1E**. Again, Spore-FP1 induced significant reduction in lung bacterial burden, compared to BCG alone (*p*=0.0111). Similarly, a significant and even greater reduction was also observed in the spleens, where in all but one animal bacteria levels were below the determination threshold (*p*=0.0001) (**Figure 1F**). This outcome substantially corroborated the reduced gross lung pathology observed in the vaccinated animals, compared to unvaccinated controls or the group that received BCG alone (**Figure 1G**). Thus, Spore-FP1 was able to significantly reduce *Mtb* burden and disease in guinea pigs, either on its own, or when used to boost BCG.

### Spore-FP1 aerosolization and NHP safety and vaccine delivery optimization studies

Spore-FP1 was aerosolized using the Omron mesh nebulizer and we first established parameters to deliver the required dose of vaccine to NHP. We observed that aerosolization of FP1 fusion protein resulted in its significant loss in the condensate, due to excessive denaturation. However, immobilizing the protein on spores fully preserved it and the protein recovery from the condensate was 94% (**Figure S2A**). To deliver the intended dose of 60 μg FP1 and 2x10^9^ spores, we established that 10 min nebulization of 10 ml vaccine formulation was sufficient, and that increasing either parameter resulted in decreased relative recovery (**Figure S2B**). Thus, for delivering a higher dose, the only parameter that varied was concentration of each component in the vaccine formulation, except PolyI:C, which was kept at a standard dose of 40 μg.

Two experiments were conducted to explore the safety and immunogenicity of the Spore-FP1 vaccine candidate in BCG vaccinated macaques. As the Spore-FP1 vaccine candidate was designed to boost mycobacteria-specific mucosal immunity, an aerosol delivery strategy using a portable handheld nebulizer was selected as an approach that has been shown capable of inducing local and systemic immune responses in macaques (4, 22, 31) and humans (32) in TB vaccination trials. In the first vaccine optimization experiment (macaque Study 1), repeat Spore-FP1 boost vaccinations were delivered by aerosol at a dose of 2x10^9^ heat inactivated *Bacillus subtilis* spores + 60 µg of the FP1 fusion protein, a dose informed by murine vaccine efficacy experiments (14). Spore-FP1 vaccine preparations also contained 40 µg of PolyI:C as an adjuvant strategy. Six adult male macaques received repeated Spore-FP1 (60ug) aerosol vaccinations thirteen and seventeen weeks after intradermal BCG vaccination (**Figure S2C**). A further three BCG vaccinated macaques that did not receive boosting vaccination served as a negative control group for comparative purposes, although statistical analyses were not applied due to the limited size of the groups. BCG and aerosol Spore-FP1 boost vaccinations were well tolerated by all animals. No adverse indicators were observed in animals that received aerosol Spore-FP1 boosting vaccination. Clinical parameters, such as body weight, temperature, peripheral lymph node size, and red cell hemoglobin concentration (Hb) remained within normal ranges (**Figure S2D**).

An IFN-γ ELISPOT assay was used to measure the frequency of mycobacterial antigen-specific cells in PBMC samples collected before and after each Spore-FP-1 vaccination. There were trends for a modest increase in tuberculin PPD- and Ag85B-specific IFN-γ SFU frequencies following Spore-FP1 aerosol vaccination (**Figure S2E**). However, as Spore-FP1 aerosol boosting vaccinations did not induce the distinct increase in IFN-γ production observed in murine immunogenicity experiments (14), a further optimization experiment was conducted to explore the influence of dose and delivery route on the immunogenicity of Spore-FP1 boosting vaccination delivered to BCG vaccinated macaques.

**Figure 2:**
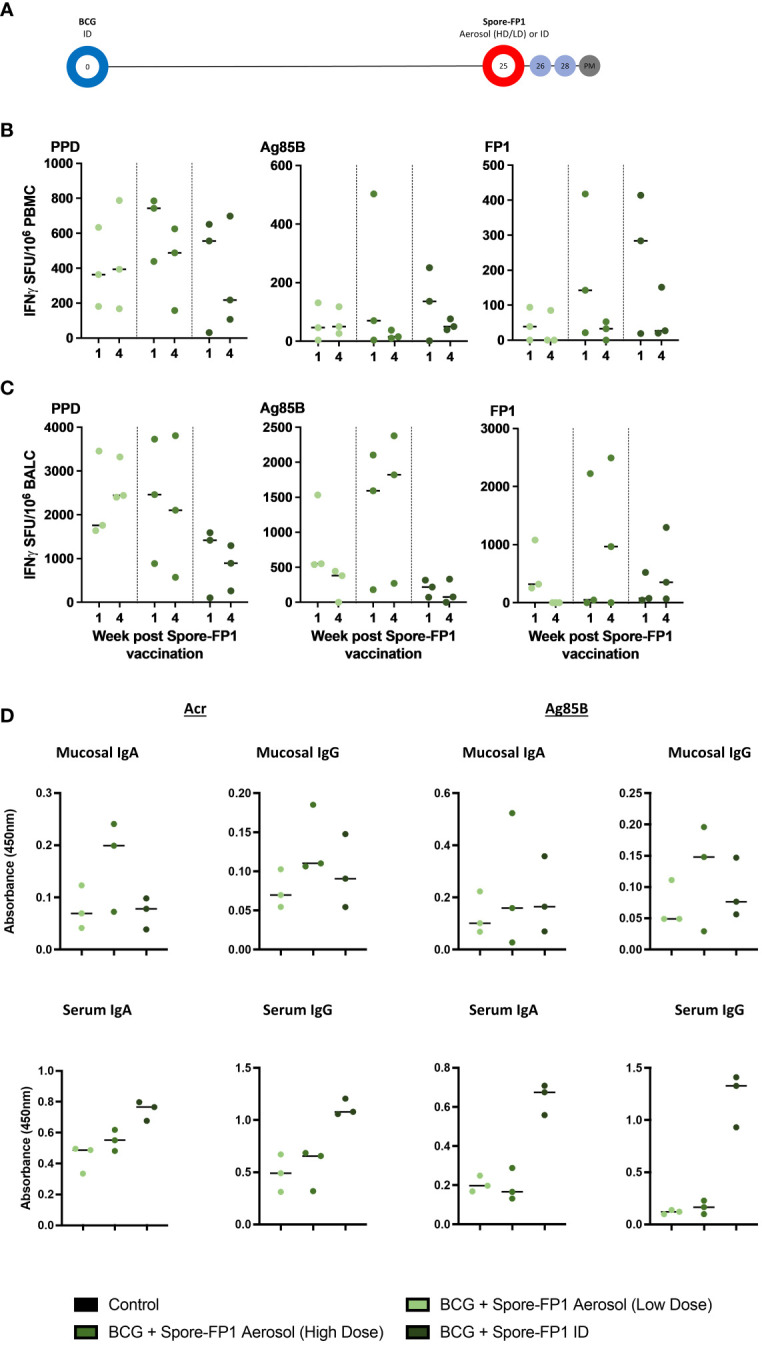
Macaque Spore-FP1 safety, immunogenicity, and vaccination optimization study. **(A)** Pilot Study 2 timeline relative to BCG vaccination. BCG immunized macaques (day 0) received either ID injection (2 x 10^9^ spores + 60 µg FP1 + 40 µg polyI:C), or low (same as ID)) or high (1 x 10^10^ spores + 300 µg FP1 + 40 µg polyI:C), dose of aerosol Spore-FP1, 25 weeks after BCG; **(B, C)** PPD, Ag85B, and FP-1 specific IFN-γ SFU measured in PBMC **(B)** and BAL cells (BALC) **(C)** samples one and four weeks after Spore-FP-1 boost vaccination. **(D)** BAL and serum IgA and IgG antibodies four weeks after Spore-FP1 vaccination (individual data points shown with median value indicated).

Hence, in the second vaccine optimization experiment (macaque Study 2), three groups of BCG vaccinated macaques (n = 3 per group) received boosting vaccination with either: a five-fold increased dose of Spore-FP1 consisting of 1x10^10^ *Bacillus subtilis* spores with 300 µg FP1 (Spore FP-1 300ug) delivered by aerosol; Spore-FP1 (60ug) also delivered by aerosol to align with the dose and route applied to macaque Study 1; or Spore-FP1 (60ug) delivered by intradermal injection to compare the immunogenicity of parenteral vaccine delivery (**Figure 2A**). Clinical parameters remained within normal ranges indicating that Spore-FP1 vaccination remained well tolerated when delivered at a higher dose by aerosol or by intradermal injection (**Figures S3A–C**). Moderate induration was observed as a local reaction at the site of Spore-FP1 intradermal injection (**Figure S3D**).

Interferon-γ ELISPOT assays were applied to PBMC, and mononuclear cells isolated from BAL samples collected one and four weeks after delivery of Spore-FP1 boost vaccination, and antigen-specific SFU compared between the groups to assess the relative immunogenicity of each vaccination regimen. Systemic frequencies of, PPD, Ag85B and FP1-specific IFN-γ producing cells were highest in the group that received the 60-µg dose of Spore-FP1 by ID injection or the 300-µg dose as an aerosol (**Figure 2B**); whereas SFU frequencies measured following lower dose (60ug) aerosol boost were comparable to those detected in macaque Study 1. Conversely, IFN-γ SFU frequencies measured in BAL tended to be higher following aerosol delivered boosting vaccination with the greatest frequencies of Ag85B- and FP1-specific SFU measured in the higher (300 µg) dose aerosol Spore-FP1 vaccinated animals (**Figure 2C**).

We also detected weak antibody responses to both Ag85B and Acr in BAL of animals receiving the high aerosol dose (**Figure 2D**). In contrast, significantly higher IgA and IgG titers to both antigens were observed in sera of animals immunized by ID Spore-FP1 over aerosol dosing (**Figure 2D**). From this pilot study, we decided to proceed with one intradermal and one aerosol-high dose boost to BCG, in the main study described below (macaque Study 3).

### Immunogenicity and efficacy of the BCG + Spore-FP1 vaccination regimen against low-dose aerosol *Mtb* challenge in macaques

#### Vaccine induced IFN-γ responses in NHPs

**Figure 3:**
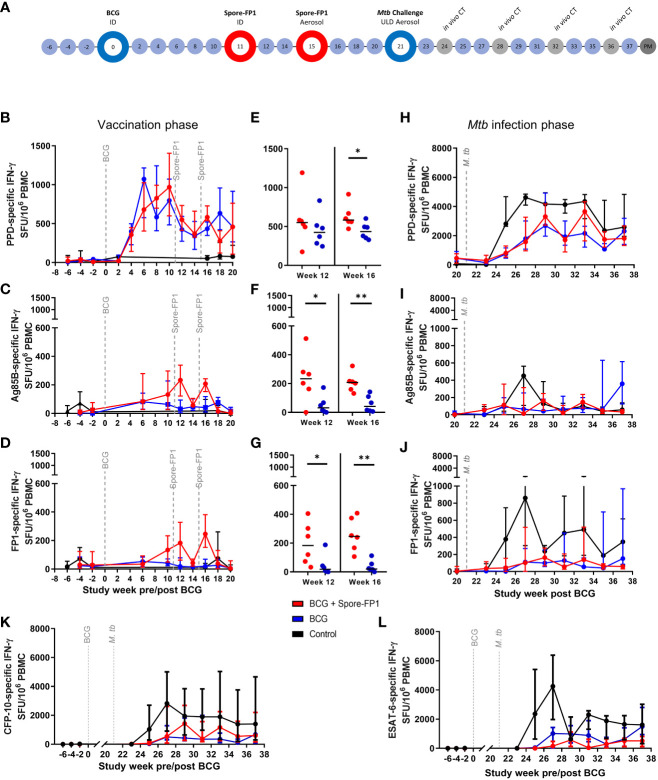
Macaque Spore-FP1 vaccine immunogenicity and efficacy against low dose aerosol *Mtb* challenge study. **(A)** Study timeline relative to ID BCG vaccination. Rhesus macaques received BCG vaccination delivered by intradermal injection at study week zero or were left as unvaccinated controls (n = 6). Spore-FP-1 boost vaccinations were delivered to six BCG vaccinated macaques by intradermal injection at study week 11 followed by a second Spore-FP-1 vaccination delivered by aerosol at study week 15 (n=6). The remaining BCG vaccinated macaques received no boosting vaccination (n = 6). All animals received low dose aerosol challenge with *Mtb* Erdman strain at study week 21 and were monitored for up to 16 weeks following infection (study week 37). Blue shaded circles represent procedures involving blood sample collection and application of immunological analyses, large open circles represent key study events: vaccination and ULD aerosol *Mtb* challenge, open circles indicate application of *in vivo* CT scanning. Animals were euthanized, and post-mortem (PM) necropsies conducted upon completion of the study schedule (shaded circle). Antigen-specific IFN-γ production measured by ELISPOT assay before and after vaccination: **(B)** PPD-, **(C)** Ag85B- and **(D)** FP-1-specific SFU following BCG vaccination and Spore-FP1 boost vaccinations. **(E)** PPD-, **(F)** Ag85B- and **(G)** FP-1-specific SFU measured at week 12 and 16 post-BCG vaccination (one week after each Spore-FP-1 boost vaccination. Antigen-specific IFNγ production measured by ELISPOT assay following *Mtb* infection: **(H)** PPD-, **(I)** Ag85B- and **(J)** FP-1-specific SFU measured following *Mtb* infection, **(K)** and **(L)** show CFP-10- and ESAT-6-specific IFNγ SFU measured before and after *Mtb* challenge. Line plots show the group median +/- interquartile range. Significant differences determined by Mann-Whitney U-Test are denoted with asterisks: p ≤ 0.05, **p ≤0.01 (all P values are unadjusted for multiple comparisons.

Informed by the findings of the macaque Spore-FP1 vaccine optimization studies (macaque Studies 1 and 2), we devised a prime-boost vaccination regimen consisting of an ID delivered primary vaccination with BCG (Danish strain 1331), followed by repeated boosting vaccinations with the Spore-FP1 candidate delivered by ID injection (LD: 2x10^9^ *B. subtilis* spores + 60 ug FP-1), and then by aerosol (HD: 1x10^10^ *B. subtilis* spores + 300 ug FP-1) at weeks 11 and 15 after BCG vaccination, respectively. Six macaques received the BCG + Spore-FP1 vaccination regimen, six received BCG vaccination alone and a further six were left as an unvaccinated control group. Twenty-one weeks after delivery of the initial BCG vaccinations, all study animals received a low-dose aerosol *Mtb* challenge (**Figure 3A**).

Vaccination with BCG and Spore FP-1 delivered by ID injection or aerosol was well tolerated with no adverse reactions apparent in clinical or behavioral parameters (**Figure S4**). Moderate skin induration was observed at the site of intradermal BCG or Spore-FP1 vaccination, similar as in Study 2, indicating the induction of an immune response locally at the site of vaccination (**Figure S4D**). Following the *Mtb* challenge, clinical parameters remained stable, with red blood cell sedimentation rate increasing only moderately in one animal in each experimental group (**Figures S4E, F**).

Vaccine induced cellular immune responses prior to *Mtb* challenge were interrogated using an *ex-vivo* IFN-γ ELISPOT (**Figures 3B–L**). PPD-specific IFN-γ SFU frequencies increased following BCG and reached a post vaccination peak between weeks 6 – 10, with little evidence of increase following the ID or aerosol Spore-FP1 vaccination at weeks 11 or 15 (**Figures 3B, E**). In contrast, Ag85B- and FP1-specific IFN-γ SFU profiles indicated that the Spore-FP1 boosting vaccinations were immunogenic when delivered by intradermal injection and by aerosol (**Figures 3C–J**), with both Ag85B- and FP1-specific IFN-γ spot forming unit (SFU) frequencies measured in PBMCs isolated from boosted animals significantly higher than in animals vaccinated with BCG alone (**Figures 3C, D, F, G**).

#### Cell subsets and cytokines

**Figure 4:**
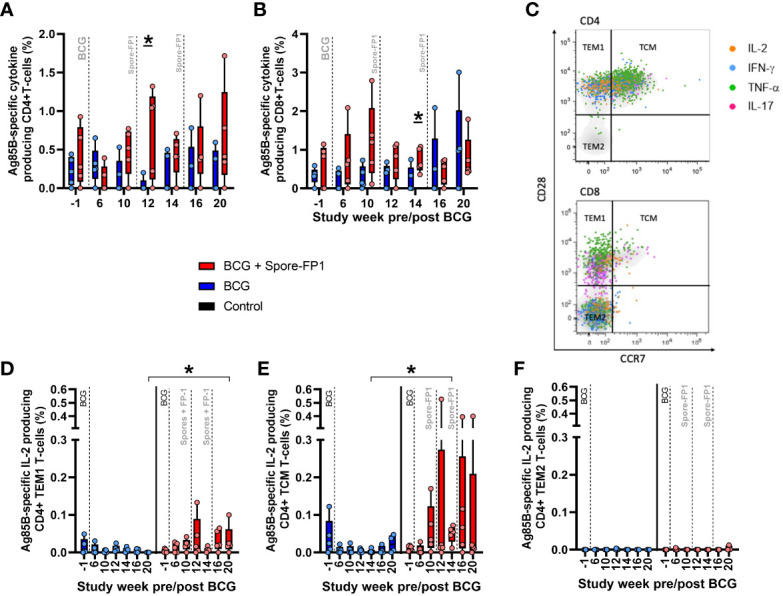
Ag85B-specific CD4 and CD8 T-cell profiles in PBMC from vaccinated macaques. Frequency of Ag85B-specific cytokine producing CD4 and CD8 T-cells measured by intracellular cytokine staining. Dots represent the summed frequency of CD4 **(A)** and CD8 **(B)** T-cells producing IFN-γ, IL-2, TNF-α or IL-17 measured in peripheral blood mononuclear cells (PBMCs) collected from individual animals prior to and following ID BCG and Spore-FP1 vaccination. Panel **(C)** representative bivariate density plots of central to effector memory T-cell populations defined by the pattern of CD28 and CCR7 staining on CD95 expressing CD4 and CD8 T-cells, overlaid with cytokine producing cells represented as colored coded dots. Frequencies of antigen-specific IL-2 producing **(D)** TEM1, **(E)** TCM and **(F)** TEM2 CD4 memory T-cells measured prior to, and following, ID BCG and Spore-Fp1 boosting vaccinations. Bars show the group median +/− IQR with minimum and maximum values indicated by box whiskers with the dots representing values measured in individual animals.

The systemic cellular immune response was further interrogated using flow cytometry based multiparameter intracellular cytokine staining (ICS) assays to measure production of the cytokines IFN-γ, IL-2, TNF-α and IL-17 from CD4 and CD8 T-cell populations in PBMCs. This analysis indicated that vaccination with Spore-FP1 increased the frequency of CD4 T-cells producing Ag85B-specific IFN-γ, IL-2, TNF-α or IL-17 relative to BCG vaccinated animals, with summed frequencies of cytokine producing cells significantly higher one week following ID Spore-FP1 vaccination, then remaining elevated following aerosol vaccination (**Figures 4A, B**). Examination of the frequency of CD4 T-cells producing individual cytokines revealed that the Ag85B-specific response was comprised of both Th1 (IFN-γ, IL-2, or TNF-α) and Th17 (IL-17) populations (**Figure S5A**). To explore the memory differentiation status of the Ag85B-specific CD4 T-cell population, cytokine producing central and effector memory populations were identified as cells expressing the activation marker CD95, followed by expression pattern of the co-stimulatory receptor CD28 and lymph node homing marker CCR7. Therefore, central to effector T-cell differentiation was determined on antigen-specific CD4 T-cells as (TCM) CD28+CCR7+; CD28+CCR7- transitional effector memory (TEM1); and fully differentiated (TEM2) CD28-CCR7- cells (**Figure 4C**). CD4 T-cells producing Ag85B-specific IFN-γ, IL-2, TNF-α, or IL-17 (summed) primarily occupied the Transitional effector memory (TEM1, CD28+ CCR7-) phenotype, or Central memory (TCM, CD28+ CCR7+) phenotype, with a non-significant trend for higher frequencies of TEM1 cells in the BCG+Spore-FP1 vaccinated group (**Figure S5C**). Comparison of IFN-γ, IL-2, TNF-α and IL-17 production individually in CD4 memory T-cell populations revealed significant differences between the BCG and BCG + Spore-FP1 vaccination groups in terms of TCM and TEM1 populations producing Ag85B-specific IL-2, at weeks 14 and 20 following BCG vaccination (three weeks after ID and aerosol Spore-FP1 boost, respectively) (**Figures 4D, E**). TEM2 phenotype Ag85B-specific CD4+ responses were not detected (**Figure 4F**). No significant differences were measured between the groups in terms of CD4 memory populations producing IFN-γ, TNF-α or IL-17 (data not shown). CD8 T-cells producing Ag85B-specific IFN-γ, IL-2, TNF-α or IL-17 were measured in BCG vaccinated and BCG+Spore-FP1 boosted animals; although summed frequencies (**Figure S5C**), and frequencies of cells producing each cytokine individually (**Figure S5B**), did not significantly differ from pre-vaccination levels, or between the vaccination groups.

#### Cellular immune responses measured following low dose aerosol challenge with Mtb

Analysis of the cellular immune response using the IFN-γ ELISPOT assay was continued following low dose aerosol challenge and expanded to include overlapping 15-mer peptide pools spanning the CFP-10 or ESAT-6 sequence to provide a measure of the *Mtb*-specific response (**Figures 3K, L**). Comparison of IFN-γ SFU values between the experimental groups indicated that CFP-10 and ESAT-6 specific IFN-γ SFU frequencies were significantly higher in unvaccinated animals relative to BCG and BCG+Spore-FP1 vaccinated groups (**Figures 3K, L**). These trends were also apparent in Ag85B-, FP1- and PPD-specific IFN-γ SFU profiles (**Figures 3H–J**).

### Antibody responses in NHP

**Figure 5:**
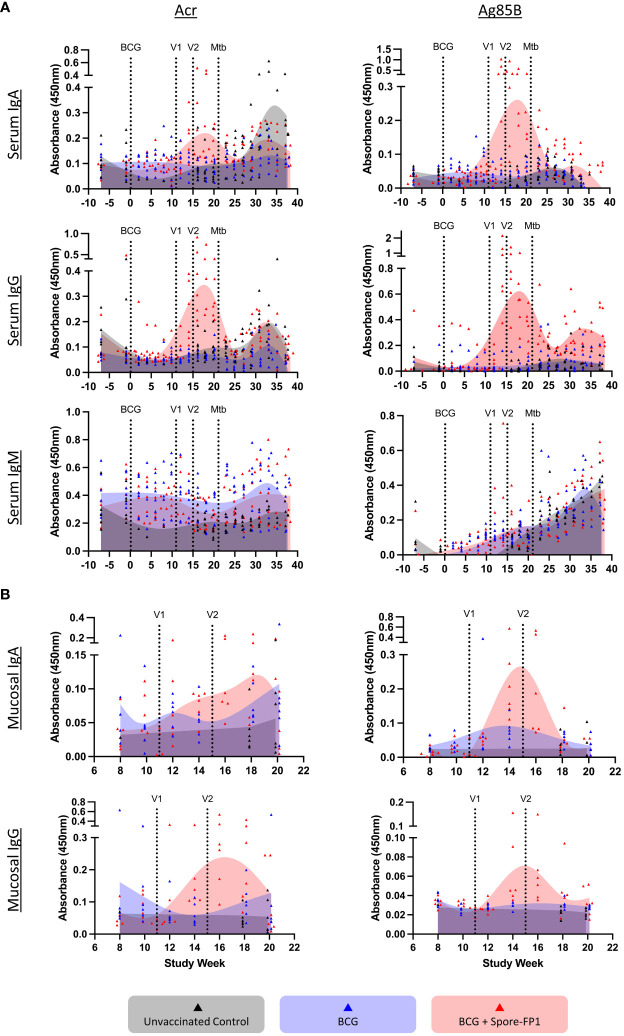
Antibody responses in immunized NHP. Blood samples were taken throughout the study protocol and nasal swabs during immunization phase only. Panel **(A)** serum (weeks -6 through 38) and **(B)** mucosal responses (weeks 8-20) for indicated antibody isotypes detected against Ag85B and Acr by antigen-specific ELISA. Individual animals are indicated by dots and the histograms represent the temporal mean responses from all animals in the three immunization groups (n=6/group), shown as smoothing spline curves fitted by GraphPad Prism. Vertical lines indicate immunizations and *Mtb* challenge.

Antibody responses were tested in sera of immunized NHPs throughout the study period, including after *Mtb* challenge, and in nasal swabs, only prior to challenge. Reactivity to the two principal antigens, Ag85B and Acr, was determined by ELISA. Following two boost vaccinations, the animals showed a small increase in circulating IgM antibodies, which steadily increased following *Mtb* challenge (**Figure 5A**). IgA responses spiked more prominently following vaccination, particularly to Ag85B, but then subsided following *Mtb* challenge. The most prominent increase was observed for IgG antibodies, for both antigens. Interestingly, the IgG responses then declined sharply before the *Mtb* challenge, and only increased transiently in some animals after *Mtb* challenge but returned to pre-boosting levels by week 45.

As for mucosal antibodies response, we observed both IgA and IgG responses to both antigens in nasal swabs, which increased following aerosol boost vaccination. These responses, however, appear short-lived and declined to pre-vaccination levels by week 20 (**Figure 5B**).

### Efficacy of spore-FP1 vaccination in NHP against low-dose aerosol challenge with *Mtb*

**Figure 6:**
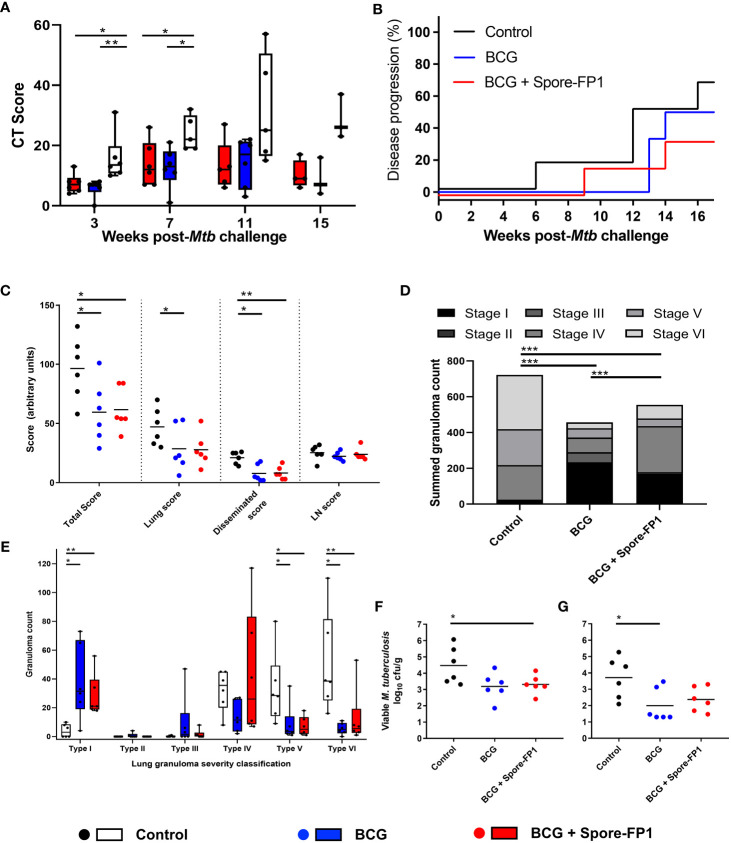
*Mtb* challenge induced disease measures in NHP. **(A)** CT Scores, lesion counts and occurrence of lymph node involvement following low dose aerosol *Mtb* challenge. Box plots show group median (+/- interquartile range) with minimum and maximum values connected by whiskers. **(B)** Kaplan–Meier plot indicating the proportion of vaccinated and unvaccinated animals that developed progressive disease that met pre-determined humane endpoint criteria following challenge with *Mtb.* **(C)** Total, pulmonary, disseminated (spleen, liver, kidneys) and lymph node (LN) associated tuberculosis-induced disease measured using a gross pathology score system. **(D)** Occurrence and severity classification (stages I–VI) of tuberculous granulomas identified by microscopic examination of representative H&E-stained lung tissue sections. Bars represent the group median +/- interquartile range with minimum and maximum values indicated by box whiskers. Dots represent granuloma counts measured in individual animals. **(E)** Total number of granulomas (stage I–VI combined) identified in the lung from representative H&E-stained sections. Stacked bars indicate the summed number of granulomas observed and the combined granuloma totals for each experimental group. **(F)** Summed viable *Mtb* recovered from tissue samples collected at necropsy and, **(G)** from extrapulmonary tissues only. Individual points represent log_10_CFU/g tissue recovered from each animal with the group median indicated by horizontal bars. Non-parametric Mann–Whitney *U* tests were used for group-wise comparisons of CT scores, pathology scores, granuloma/lesion counts and viable CFU counts recovered from tissues, with unadjusted results reported as: *P ≤ 0.05; **P ≤ 0.01; ***P ≤ 0.001. Cochran–Armitage method *X* ^2^ tests were used to compare summed granuloma stage scores between groups, unadjusted results are reported as *P ≤ 0.05; **P ≤ 0.01; ***P ≤ 0.001.

A range of clinical and behavioral parameters were used to assess disease progression following *Mtb* exposure (**Figures 6**, **S6**, **7**). The development of disease was monitored using *in vivo* CT scanning applied three, seven, 11 and 15 weeks after aerosol *Mtb* challenge and disease burden evaluated using a quantitative score system based on the extent and characteristics of disease present (26). The CT scores recorded from the BCG and BCG + Spore FP1 vaccinated groups were significantly lower than in the unvaccinated control animals three and seven weeks after *Mtb* challenge (**Figure 5A**). These differences were driven in part by significantly higher number of lesions quantified in the CT scans collected from this group seven weeks after *Mtb* challenge (**Figure S6A**) and significantly greater disease observed in the lymph nodes of unvaccinated animals three weeks post infection (**Figure S6B**).

Where disease burden developed to meet pre-determined humane end-point criteria, animals were euthanized prior to completion of the experimental schedule. The occurrence of disease progression did not differ significantly between the experimental groups, with 66.6% of the unvaccinated, 50% of the BCG vaccinated and 33.3% of the Spore-FP1 vaccinated animals developing disease levels that met humane end-point criteria (Log-rank *p = 0.47*) (**Figure 5B**).

A full post-mortem examination was conducted, and tuberculous disease pathology assessed in a range of pulmonary and extra-thoracic tissues using a gross pathology scoring system developed for assessment of the occurrence and extent of tuberculous disease (5). Tissue samples were collected from a range of extra-pulmonary tissues for quantification of viable *Mtb* by bacterial culture as well as representative samples which were immersed into neutral buffered formalin (NBF) for histopathological analysis. The lung and heart were collected whole and fixed in NBF for *ex vivo* magnetic resonance imaging (**Figure S7**) before sectioning for microscopic histological analysis. A significant reduction in total (summed) pathology scores (*p = 0.041, p = 0.024*), and pathology scores recorded in extra-thoracic tissues (*p = 0.017, p = 0.009*), were measured in the BCG vaccinated and BCG+Spore-FP1 vaccinated groups relative to unvaccinated control animals (**Figure 6C**). A further significant reduction in lung pathology was measured in BCG+Spore-FP1 vaccinated animals relative to the unvaccinated control group (*p = 0.048*), but the outcomes between the two vaccinated groups were not significantly different (**Figure 6C**).

### Histopathological findings

Fixed tissue sections were prepared from defined regions of each lung lobe and stained with hematoxylin and eosin (H&E) for microscopic analysis. Lung granulomas were quantified and classified according to severity as: unorganized (Types I-II) and organized (Types IV-VI), as described by Rayner et al. (29) (**Figures 5D, E**). Comparison of summed granuloma severity scores between the experimental groups indicated that vaccination with BCG and BCG+ Spore-FP1 vaccination were associated with a reduced frequency of more severe lesions (types IV – VI). This was confirmed by comparison of granuloma counts recorded in lung tissue sections prepared from each animal which indicated that significantly increased frequencies of early (Type I) lesions were present in vaccinated animals, whereas significantly more Type V & VI granulomas were recorded in unvaccinated animals (**Figure 5E**).

### Viable *Mtb* recovered from tissues at necropsy

Tissue samples were collected at necropsy from hilar lymph nodes, spleen, liver, and left and right kidneys for quantification of viable *Mtb* colony forming units by bacterial culture on Middlebrook 7H11 agarose plates. Viable *Mtb* CFU were calculated per gram of tissue and summed to provide a total CFU/g value for comparison between the experimental groups (**Figure 5F**). Due to the large and consistent number of CFU typically recorded from hilar lymph node samples, these values were excluded to produce an extra-thoracic tissue viable *Mtb* CFU/g (disseminated) count (**Figure 5G**). Viable *Mtb* recovered from tissue samples collected from BCG vaccinated animals were significantly lower than from samples collected from the unvaccinated control group. Significant differences between the *Mtb* CFU recovered from BCG+Spore-FP1 vaccinated and unvaccinated animals, or those that received BCG vaccination alone, were not detected, although a general trend of one log_10_ reduction in both vaccinated groups was observed (**Figure 5G**).

### Passive transfer of NHP antibodies to mice

As we have a particular interest in antibody-mediated protection in TB as a form of immunotherapy (33–36), we also wanted to see if the antibodies induced by vaccination in macaques could impact the infection when delivered directly to the lungs. We therefore purified antibodies from pooled NHP sera from the efficacy study, using an FP1 affinity chromatography (**Figure S8A**). The purified antibodies included IgG, IgA, and IgM isotypes to both Ag85B and Acr antigens. (**Figure S8B**). Before transferring purified specific or non-specific NHP antibodies (purified by protein G chromatography from BCG-immunised animals) to mice transgenic for human high affinity IgG receptor (CD64 Tg mice), we wanted to establish whether NHP IgG binds efficiently to human IgG receptor CD64. Using serial dilutions of antibodies in a Biacore assay, we demonstrated that they indeed do, albeit with a reduced affinity of Kd=1.62x10^-7^ M, compared to Kd=2.19x10^-8^ M, for human IgG (**Figure S8C**). As we previously established that mucosal immunotherapy with antibodies works best in synergy with IFN-γ, we transferred antigen-specific or naïve NHP antibodies to CD64 Tg mice together with mouse IFN-γ, in the form of three intranasal inoculations, 2 h before, and 2 and 7 days after *Mtb* challenge (**Figure S8D**). One week after the final treatment, mice were euthanized and the bacterial burden in their lungs determined. As can be seen in **Figure S8E**, mice that received naïve IgG+IFN-γ displayed a significant reduction of lung CFU from 5.69 (95% CI 5.54 - 5.85) to 5.34 (95% CI 5.22 - 5.47). A further 4-fold reduction (4.94 (95% CI 4.76 - 5.13) could be observed in mice that received antigen-specific antibodies, thus indicating that vaccine-induced antibodies when delivered intranasally to mice did possess some protective potential in the mouse CD64 Tg model.

## Discussion

In our previous studies in mice, we demonstrated the protective potential of the Spore-FP1 vaccine candidate as a mucosal boost to BCG (14, 15). In the present study, we have further evaluated its potential in guinea pigs and NHP, as well as in mice in the form of passive (antibody) vaccination.

NHP are widely considered to be highly reflective of human TB infection and disease and are therefore the most relevant animal model for testing new TB vaccine candidates. However, NHP are also a stringent and challenging model in terms of requirements and resources, and only a small proportion of preclinical vaccine candidates are evaluated in this model. Several NHP TB vaccine studies have been performed in the last few years, mostly in highly susceptible rhesus macaques (23), with various outcomes. Thus, BCG has been consistently shown to be protective in NHP, with the degree of protection dependent on the route of administration. Typically, intradermal injection of BCG leads to a moderate reduction of lung pathology and bacterial burden but does not eliminate infection or prevent disease following *Mtb* challenge (1, 3–5, 37, 38). Interestingly, respiratory (aerosol or intratracheal) delivery of BCG in NHP was shown to be superior to intradermal injection in some studies (3, 4, 30) but not others (1). Most interestingly though, intravenous injection of BCG was shown in recent studies to be superior to all other routes, conferring a much greater degree of protection and ultimately prevention of TB disease in some animals (1, 2). While intravenous injection is not a practical or feasible route of vaccine delivery, these important studies have set a new precedent for TB vaccine induced protection in NHP and correlates associated with it.

Of the new TB vaccine candidates, the outcomes in the NHP model of infection have been more divergent. Thus, two attenuated strains of *Mtb*, namely MTBVAC (5) and *MTB* δsigH (39) conferred protection comparable to or better than BCG. Likewise, a cytomegalovirus (CMV) based vaccine candidate induced long term effector memory T cells and provided sustained protection against *Mtb* challenge in vaccinated NHP (40). This protection was underscored by absence of clinical TB disease in 40% of vaccinated animals, with some animals returning no culturable *Mtb* in their organs (40). To date, the CMV construct expressing *Mtb* antigens remains the only reported subunit TB vaccine candidate to induce protection in NHP. In contrast, several subunit TB vaccine candidates have failed to demonstrate protective potential in the NHP model of *Mtb* infection (37, 38, 41), underpinning the stringency of this model and the challenges in rational TB vaccine design, in general.

In the current study, Spore-FP1 conferred protection in guinea pigs, whether as a standalone vaccine or as a mucosal BCG-boost vaccine. The level of reduction of the bacterial burden in naïve guinea pigs was inferior to BCG but a statistically significant 7-fold reduction, compared to mock immunized animals. In BCG primed animals, this was even more substantial, with a further near 100-fold reduction in the lungs compared to BCG alone, and no detectable bacteria in all but one animal in the spleen. This was also corroborated by a visible improvement of gross lung pathology, with fewer granulomatous surface areas than in BCG or mock immunized animals. Historically, the guinea pig model of *Mtb* infection has been shown to reflect the human TB disease pathology better than mice (42) but also proven difficult to improve protection upon BCG (43), whether as prime or boost, and only a small number of vaccine candidates have been shown to prolong animal survival (44–46), or reduce pulmonary lesions (47), but not the bacterial load in organs. Thus, our data from this animal model of infection signify the protective potential of Spore-FP1 as a promising TB vaccine candidate, which justified its further testing in the NHP model.

The macaque immunogenicity and efficacy studies described herein have demonstrated that boosting vaccination with the Spore-FP1 candidate was well tolerated by BCG primed rhesus macaques, with no adverse clinical or behavioral indications observed following ID injection with 2 x 10^9^ spores + 60 µg FP1, or aerosol delivery of 1 x 10^10^ spores + 300 µg FP1, in both instances combined with 40 µg PolyI:C adjuvant. To our knowledge, this is the first application of a vaccine candidate employing heat inactivated *Bacillus subtilis* spores as an antigen delivery system, or PolyI:C as a mucosal adjuvant, to a non-human primate model. Therefore, the absence of adverse reactions is an observation important for the future development of vaccines using the heat inactivated spore delivery system or PolyI:C adjuvant for systemic or mucosal delivery.

Immune profiles measured in peripheral blood mononuclear cells following vaccination with Spore-FP1 indicated that the vaccine candidate was immunogenic when delivered by ID injection, or as an aerosol, and induced a significant increase in Ag85B-specific cellular immune responses including IFN-γ secreting cells measured by ELISPOT, and CD4 T-cells with a Th1 and Th17 functional profile measured by flow cytometric ICS assay. The memory differentiation profile of antigen-specific CD4 T-cells was similar between the Spore-FP1 boosted and the group that received BCG vaccination alone. However, the significant increase in IL-2 producing TCM cells measured three weeks after ID Spore-FP1 vaccination, and IL-2 producing TEM1 cells measured three weeks after aerosol delivery of Spore-FP1, may indicate increased proliferation of boost vaccination-induced central memory cells and subsequent differentiation and proliferation of effector populations following repeat delivery and immunological exposure to the vaccine antigens. Interestingly, IFN-γ ELISPOT responses were lower in both vaccinated groups than in the control group during the *Mtb* infection phase, and we speculate that this could be associated with the level of infection, which drives the effector T cell responses, rather than the vaccine induced TCM.

The efficacy of the Spore-FP1 boosting vaccination regimen against low dose aerosol challenge with *Mtb* Erdman strain was assessed by comparison of a range of clinical, gross and microscopic pathology measures, and the quantification of viable *Mtb* bacteria in lungs and extra-pulmonary tissues. These analyses indicated that the extent of tuberculous disease pathology measured in lung and extra-pulmonary tissues using both macro- and microscopic methodologies was significantly reduced in the Spore-FP1 boosted animals in comparison to the unvaccinated group but were equivalent to animals receiving BCG vaccination alone. Similarly, microbiological analyses revealed a significant reduction in viable *Mtb* CFU recovered from lung-associated or extra-pulmonary tissue samples of BCG vaccinated animals in comparison to the unvaccinated control group, whereas a non-significant trend for reduction was apparent between Spore-FP1 boosted and unvaccinated animals, with numbers of *Mtb* CFU effectively equivalent between the vaccinated groups. Overall, these findings indicate that the Spore-FP1 vaccination regimen assessed in this experiment was effective at inducing systemic cellular immune responses to the Ag-85B and FP-1 vaccine antigens and whilst it led to significant reduction in TB-induced disease relative to unvaccinated individuals, it did not improve upon the efficacy afforded by BCG vaccination alone against low dose aerosol challenge with *Mtb*.

Our choice of one intradermal and one aerosol boost with Spore-FP1 for NHP was based on the pilot experiments which indicated that a high aerosol dose was required to achieve similar levels of antigen-specific IFN-γ induction in peripheral blood cells to that seen by intradermal injection. The ‘high’ aerosol dose was 5-fold higher than the intradermal dose, or indeed the originally intended aerosol dose. This was necessitated by logistical constraints of aerosolized delivery in this model, whereby the anesthetized animals are exposed to the aerosol for up to ten minutes through a mask, while breathing freely. As this is not a hermetically closed system, controlling the aerosolized vaccine intake in this way is difficult, and our subsequent *in vitro* simulation studies indicated that only a small proportion of the vaccine might have been delivered to the lungs. This limitation is exacerbated by the fact that, unlike live replicating vaccines such as BCG or viral vectors, where dose may not be as critical, a protein-based vaccine such as Spore-FP1 is more likely to be dose dependent for efficacy. To our knowledge, this was only the second attempt of aerosolized protein-based TB vaccine in NHP, following the recent study by Darrah et al. (38), in which the recently trialed-in-humans M72 (8) and H56 (48) protein+adjuvant vaccines were delivered to NHP simultaneously by intramuscular and aerosol route. Interestingly, despite inducing local (lung) and systemic immune responses, neither vaccine could improve protection afforded by BCG when used to boost it (38). In that study, no details were given about protein recovery following the nebulization process. However, the nebulization process may be harmful to soluble proteins, more so than BCG or replicating viral vectors, as the forces involved can lead to protein denaturation and aggregation. Indeed, our experiments with Spore-FP1 indicated that the fusion protein could not be recovered from the aerosol condensate when nebulized on its own, due to denaturation and precipitation in the nebulizer. However, adherence to spores protected the protein during the process, so that it could be recovered from the surface of spores in condensate without a significant loss. Alternatively, where proteins cannot be immobilized, we found previously that addition of 0.1% Polysorbate 80 stabilizes the protein during aerosolization and preserves its biological function (35).

Despite the failure to confer protection by active vaccination in NHP, we set out to test the protective potential of Spore-FP1 induced antibodies, by transferring them to transgenic mice expressing human high affinity Fcγ receptor (CD64). Following transfer to human CD64 transgenic mice, antibodies (containing predominantly IgG and some IgA) from BCG immunized NHP, when combined with IFN-γ, reduced *Mtb* infection by approximately 5-fold, in a statistically significant manner. However, FP1 antigen-specific antibodies from vaccinated animals (mixture of anti-Ag85B and Acr antibodies), induced a further statistically significant 4-fold reduction of lung CFU, compared to BCG induced antibodies. It would have been interesting to also test in this model the mucosal antibodies from Spore-FP1 immunized NHP, but we were not able to purify enough for the passive transfer.

In conclusion, we evaluated the protective potential of the Spore-FP1 vaccine candidate, previously shown to be protective in mice, in the two more advanced animal models, namely guinea pigs and NHP. Intranasal vaccination with Spore-FP1 conferred protection in both naïve and BCG primed guinea pigs, as demonstrated by significantly reduced bacterial counts in the lungs and spleens, and reduced lung pathology. Spore-FP1 was well tolerated in NHP, when delivered either by the intradermal or aerosol route, but despite inducing systemic and mucosal immune responses, it fell short of adding to the protection afforded by BCG, either in terms of lung pathology or bacterial burden. These findings highlight the challenges in reconciling disparate outcomes for TB vaccine-induced protection in different animal models and underlie the need for further improvement in both vaccine formulations and the capability to deliver controlled vaccine doses by the respiratory route.

## Data availability statement

The original contributions presented in the study are included in the article/**Supplementary Material**. Further inquiries can be directed to the corresponding authors.

## Ethics statement

'The animal studies in this publication were approved by the UKHSA Establishment Animal Welfare and Ethical Review Committee and authorised under a UK Home Office project licence. For the study conducted at the Karolinska Institute, authorisation was from the Stockholm Animal Research Ethics Committee (no. 210-14). All studies were conducted in accordance with local legislation and institutional requirements.

## Author contributions

ADW oversaw the macaque studies, analyzed the data, and co-wrote the manuscript; AT performed the antibody analysis and passive transfer to mice; LS, CS, AM, SL, FL, SC, ADW, SS, and MD performed procedures, acquired, analyzed, and interpreted macaque study data. FS and ER prepared, acquired and interpreted histopathology materials and performed pathology analyses for studies at UKHSA; FG interpreted and analyzed macaque radiography images; SC, SZ, MER, and JIB performed the guinea pig studies; AC, PH, GD, MK, and MP were involved with vaccine formulation, aerosolization and optimization studies; MSi and MSt provided vaccine antigens; SMC provided HU58 spores; AW advised on guinea pig studies at UKHSA; SS, MD, and ADW conceived the studies with RR, and contributed to the design of macaque experiments and manuscript preparation; RR developed the Spore-FP1 vaccine formulation, participated in all experimental designs and co-wrote the manuscript. All authors contributed to the article and approved the submitted version.

## Supplementary material

Supplementary Figure 1 Example flow cytometric gating strategy. Sequential gating used for cell population analysis described in manuscript **Figure 3** and **supplementary Figure S5**.

Supplementary Figure 2 Aerosolization of Spore-FP1 and macaque safety and immunogenicity Study 1 **(A)** Coomassie Blue stained gel of FP1 fusion protein following nebulization and recovery from condensate; Lanes: 1. Protein standards; 2. FP1 alone before nebulization, 5 μg; Lanes 3-5. FP-1 after nebulization, equivalent to 5. 3 and 1 μg, respectively; 6. Equivalent to 5 μg of FP-1 recovered from spores after nebulization; 7. Protein eluted from empty spores after nebulization. **(B)** Effect of nebulization time and sample volume on recovery of the FP1 protein. **(C)** Pilot Study 1 timeline relative to BCG vaccination. Rhesus macaques (*n* = 9) received BCG vaccination delivered by intradermal (ID) injection at study week zero. Spore-FP1 boosting vaccinations at a dose of 60 ug/ml (LD) were delivered by aerosol (n = 6) at weeks 13 and 17. No boost vaccination was applied to the remaining three animals. Blue shaded circles represent procedures involving blood sample collection and application of immunological analyses, large open circles represent key study events: BCG vaccination and Spore-FP-1 boost vaccinations. All animals were euthanized, and post-mortem (PM) necropsies conducted upon completion of the study schedule (shaded circles). **(D)** clinical parameters (weight, temperature, and red blood cell hemoglobin concentration) following Spore-FP1 aerosol vaccination; **(E)** PPD, Ag85B, and FP-1-specific IFN-γ spot forming units (SFU) were measured in PBMC samples prior to and at regular intervals following each boost vaccination. Note, not all samples were available for the analysis in panel **(E)**.

Supplementary Figure 3 Macaque pilot vaccination optimization Study 2. Clinical parameters measured before and after Spore-FP1 boost vaccination delivered by high (HD) or low dose (LD) aerosol or intradermal injection (ID). **(A)** body weight; **(B)** body temperature; **(C)** red blood cell hemoglobin concentration. Box plots show median values for each vaccination group with minimum and maximum values, symbols represent data measured from individual animals. **(D)** Group median (+/- interquartile range) local skin reaction size measured following ID Spore-FP1 vaccination (n=3/group).

Supplementary Figure 4 Vaccine efficacy evaluation – main study. Clinical parameters measured throughout the vaccination and challenge phases of the experiment. **(A)** body weight; **(B)** body temperature; **(C)** red blood cell hemoglobin concentration; **(D)** local skin reaction size measured following ID BCG (left panel, two vaccine groups) and ID Spore-FP1 (right panel) vaccination (Group median +/- interquartile range); **(E-G)** erythrocyte sedimentation rate measured in individual animals following *Mtb* infection. Box plots show the median result for the vaccination group with minimum and maximum values, symbols represent data measured from individual subjects (n=6/group).

Supplementary Figure 5 Ag85B-specific CD4 and CD8 memory T-cell profiles measured in PBMCs. Plots show the vaccination group median frequency of CD4 T-cells **(A)** and CD8 T-cells **(B)** producing the cytokines IFN-γ, IL-2, IL-17 or TNF-α.c) Summed Ag85B-specific CD4 and CD8 memory T-cell subset profiles. Responses were measured prior to (week -1) and following (weeks 6, 10, 12, 14, 16 and 20) BCG vaccination. Bars show the group medians +/− IQR with minimum and maximum values indicated by box whiskers with the dots representing the summed frequency. Significant differences between groups (Mann–Whitney U test) are indicated by bars and asterisks: *P ≤ 0.05 (all P values are unadjusted for multiple comparisons).

Supplementary Figure 6 Lesion counts **(A)** and occurrence of lymph node involvement **(B)** following low dose aerosol *Mtb* challenge. Box plots show group median (+/- interquartile range) with minimum and maximum values connected by whiskers, unadjusted Mann-Whitney U-test comparison results are reported as: **P* ≤ 0.05; ***P* ≤ 0.01; ****P* ≤ 0.001.

Supplementary Figure 7
*Ex-vivo* pulmonary MRI scans. Red outlines indicate animals in which disease progressed to meet humane end-point criteria.

Supplementary Figure 8 Purification of FP1-specific antibodies from immunized NHP and passive transfer to mice; **(A)** SDS-PAGE analysis of FP1-specific antibodies under reducing (R) or non-reducing (NR) conditions, in comparison to protein G purified fraction of nonimmune human serum; lanes: 1. Standards, 2. NHP Ig (NR), 3. NHP Ig (R), 4. Human serum IgA (NR), 5. Human serum IgA (R), 6. Human IgG (NR), 7. Human IgG (NR). **(B)** isotype analysis of purified antigen-specific Abs by ELISA (5μg/ml, with 3-fold serial dilutions); **(C)** Biacore measurement of binding of NHP and human IgG to human high affinity IgG receptor (CD64); shown are concentration dependent response curves, from which an average affinity constant could be deduced for each of the two species. **(D)** Schematic showing passive transfer of Spore-FP1 immune or BCG-immune NHP antibodies to CD64 Tg mice, followed by *Mtb* challenge; **(E)** Lung CFUs from control (untreated, n=7) or treated mice, receiving either BCG-immune NHP IgG + IFN-γ (n=6), or immune (Spore-FP1) antibodies + IFN-γ (n=6). Statistical analysis: One-way ANOVA with Tukey’s post-test.
