# Supplementary material for: Spore-FP1 tuberculosis mucosal vaccine candidate is highly protective in guinea pigs but fails to improve on BCG-conferred protection in non-human primates
Source: Front Immunol. 2023 Oct 10;14:1246826. doi: 10.3389/fimmu.2023.1246826 (PMC10594996; doi:10.3389/fimmu.2023.1246826)
Supplement: Supplementary Figure 1 — Example flow cytometric gating strategy. Sequential gating used for cell population analysis described in manuscript Figure 3 and supplementary Figure S5 . [file Image_1.pdf]

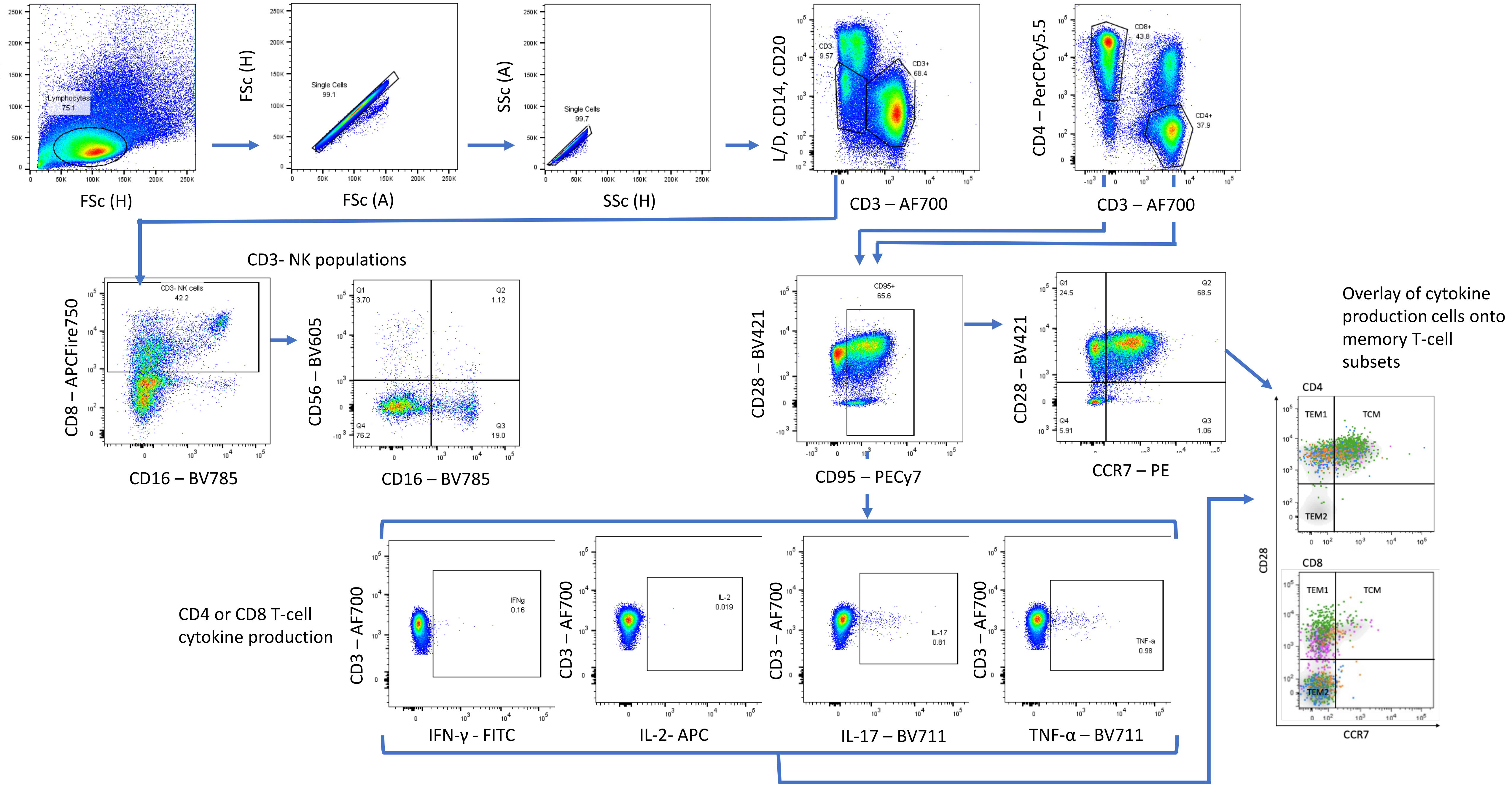
Lymphocytes
75.1
FSc (H)
Single Cells
99.1
FSc (H)
FSc (A)
Single Cells
99.7
SSc (A)
SSc (H)
CD3-
9.57
CD3+
68.4
L/D, CD14, CD20
CD3 – AF700
CD8+
43.8
CD4+
37.9
CD4 – PerCPCy5.5
CD3 – AF700
CD3- NK populations
CD3- NK cells
42.2
CD8 – APCFire750
CD16 – BV785
Q1
3.70
Q2
1.12
Q4
76.2
Q3
19.0
CD56 – BV605
CD16 – BV785
CD95+
65.6
CD28 – BV421
CD95 – PECy7
Q1
24.5
Q2
68.5
Q4
5.91
Q3
1.06
CD28 – BV421
CCR7 – PE
Overlay of cytokine production cells onto memory T-cell subsets
CD4
TEM1
TCM
TEM2
CD8
TEM1
TCM
TEM2
CD28
CCR7
CD4 or CD8 T-cell cytokine production
IFNg
0.16
CD3 – AF700
IFN-γ - FITC
IL-2
0.019
CD3 – AF700
IL-2- APC
IL-17
0.81
CD3 – AF700
IL-17 – BV711
TNF-a
0.98
CD3 – AF700
TNF-α – BV711
