# Supplementary figures and images for: Spore-FP1 tuberculosis mucosal vaccine candidate is highly protective in guinea pigs but fails to improve on BCG-conferred protection in non-human primates
Source: Front Immunol. 2023 Oct 10;14:1246826. doi: 10.3389/fimmu.2023.1246826 (PMC10594996; doi:10.3389/fimmu.2023.1246826)

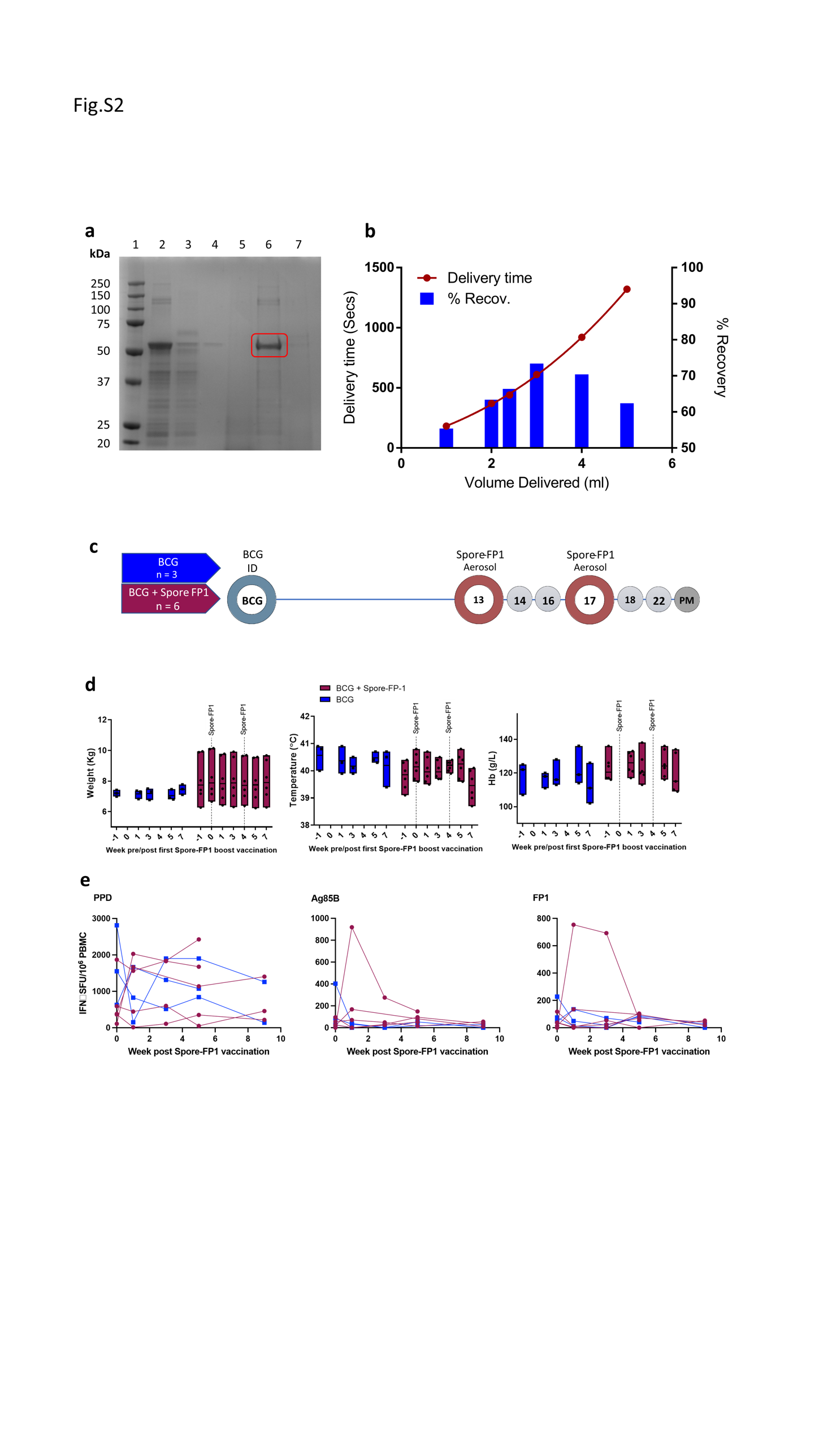

Supplement: Supplementary Figure 2 — Aerosolization of Spore-FP1 and macaque safety and immunogenicity Study 1 (A) Coomassie Blue stained gel of FP1 fusion protein following nebulization and recovery from condensate; Lanes: 1. Protein standards; 2. FP1 alone before nebulization, 5 μg; Lanes 3-5. FP-1 after nebulization, equivalent to 5. 3 and 1 μg, respectively; 6. Equivalent to 5 μg of FP-1 recovered from spores after nebulization; 7. Protein eluted from empty spores after nebulization. (B) Effect of nebulization time and sample volume on recovery of the FP1 protein. (C) Pilot Study 1 timeline relative to BCG vaccination. Rhesus macaques (n = 9) received BCG vaccination delivered by intradermal (ID) injection at study week zero. Spore-FP1 boosting vaccinations at a dose of 60 ug/ml (LD) were delivered by aerosol (n = 6) at weeks 13 and 17. No boost vaccination was applied to the remaining three animals. Blue shaded circles represent procedures involving blood sample collection and application of immunological analyses, large open circles represent key study events: BCG vaccination and Spore-FP-1 boost vaccinations. All animals were euthanized, and post-mortem (PM) necropsies conducted upon completion of the study schedule (shaded circles). (D) clinical parameters (weight, temperature, and red blood cell hemoglobin concentration) following Spore-FP1 aerosol vaccination; (E) PPD, Ag85B, and FP-1-specific IFN-γ spot forming units (SFU) were measured in PBMC samples prior to and at regular intervals following each boost vaccination. Note, not all samples were available for the analysis in panel (E). [file Image_2.tif]

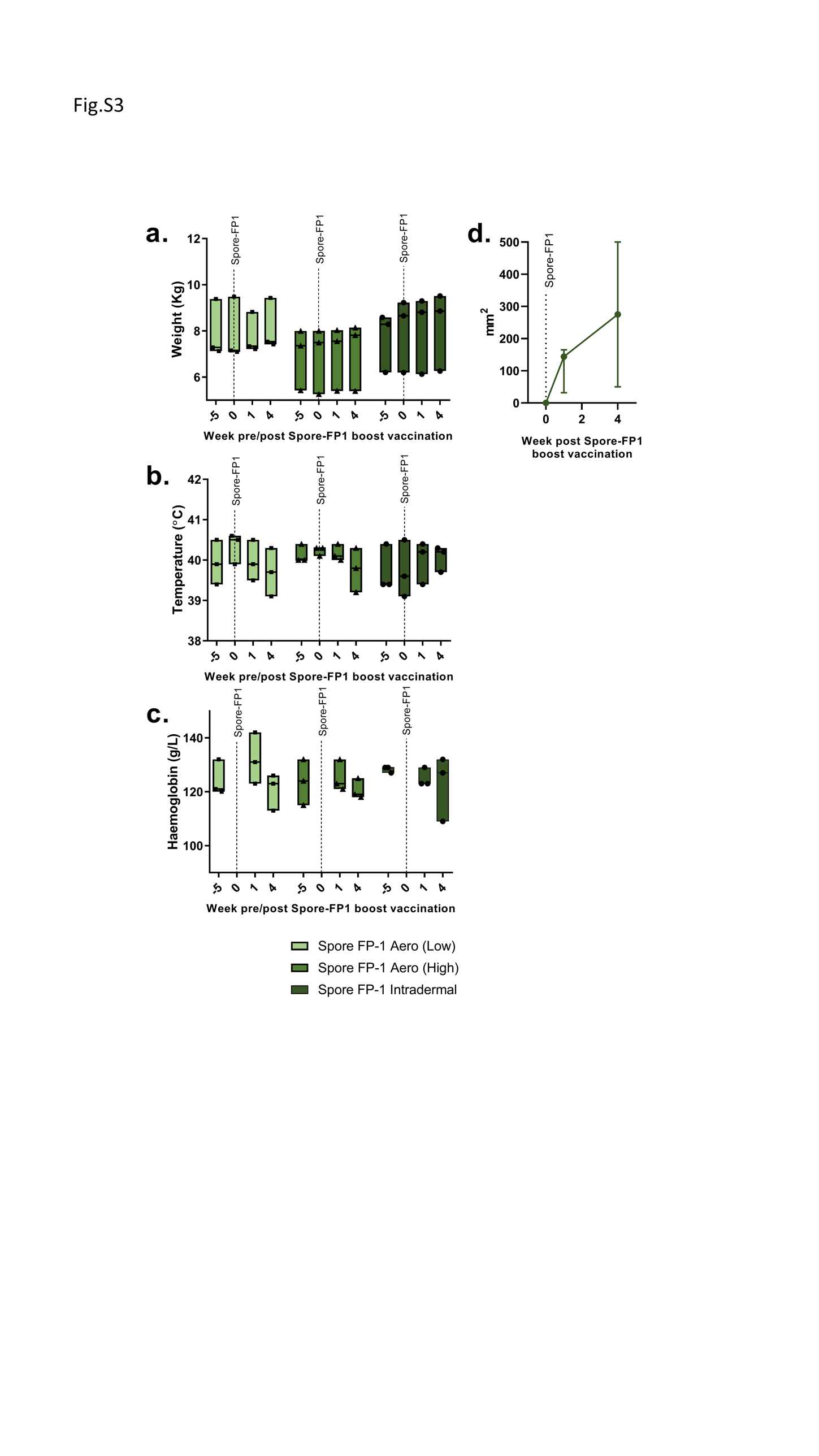

Supplement: Supplementary Figure 3 — Macaque pilot vaccination optimization Study 2. Clinical parameters measured before and after Spore-FP1 boost vaccination delivered by high (HD) or low dose (LD) aerosol or intradermal injection (ID). (A) body weight; (B) body temperature; (C) red blood cell hemoglobin concentration. Box plots show median values for each vaccination group with minimum and maximum values, symbols represent data measured from individual animals. (D) Group median (+/- interquartile range) local skin reaction size measured following ID Spore-FP1 vaccination (n=3/group). [file Image_3.tif]

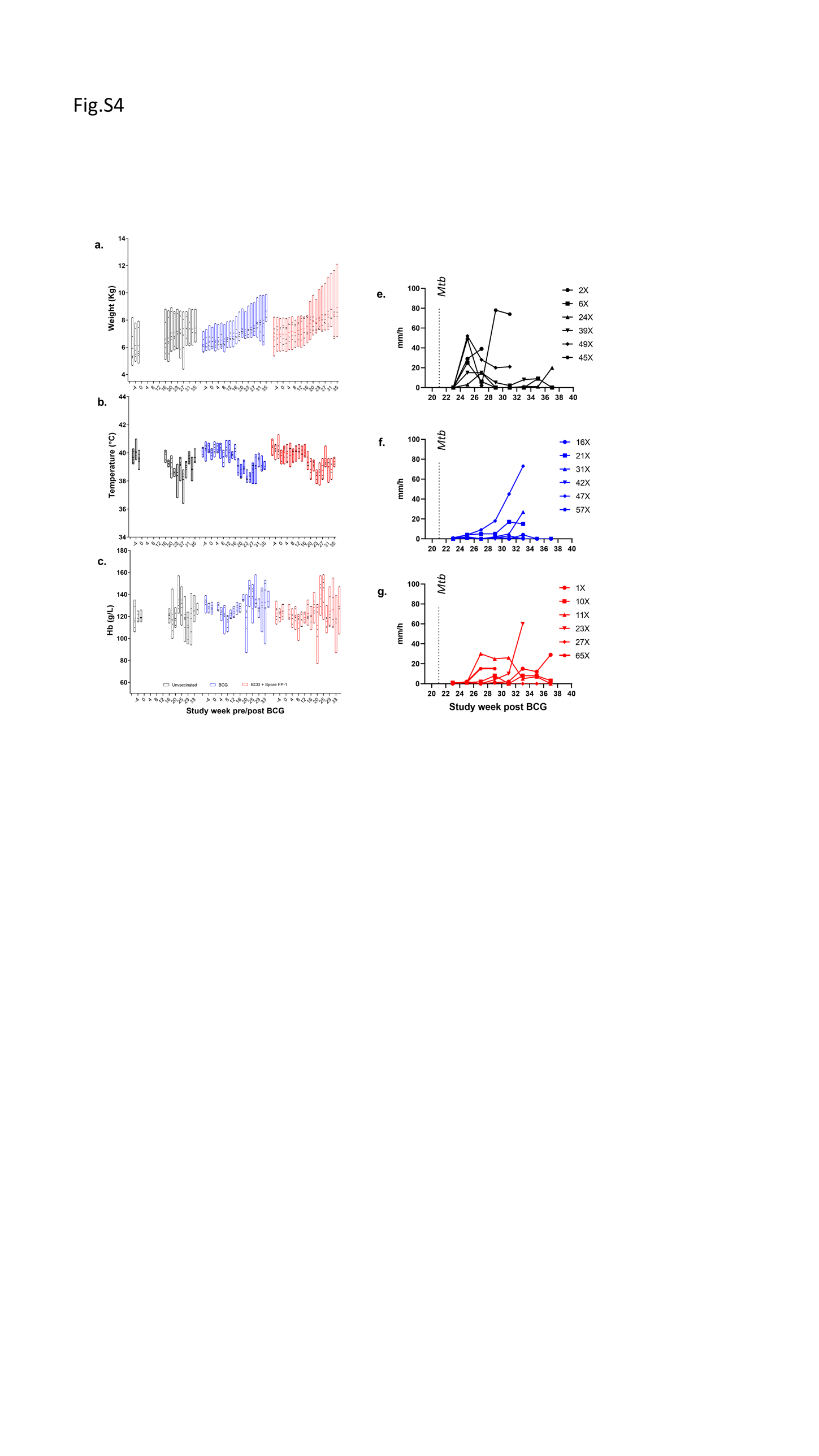

Supplement: Supplementary Figure 4 — Vaccine efficacy evaluation – main study. Clinical parameters measured throughout the vaccination and challenge phases of the experiment. (A) body weight; (B) body temperature; (C) red blood cell hemoglobin concentration; (D) local skin reaction size measured following ID BCG (left panel, two vaccine groups) and ID Spore-FP1 (right panel) vaccination (Group median +/- interquartile range); (E-G) erythrocyte sedimentation rate measured in individual animals following Mtb infection. Box plots show the median result for the vaccination group with minimum and maximum values, symbols represent data measured from individual subjects (n=6/group). [file Image_4.tif]

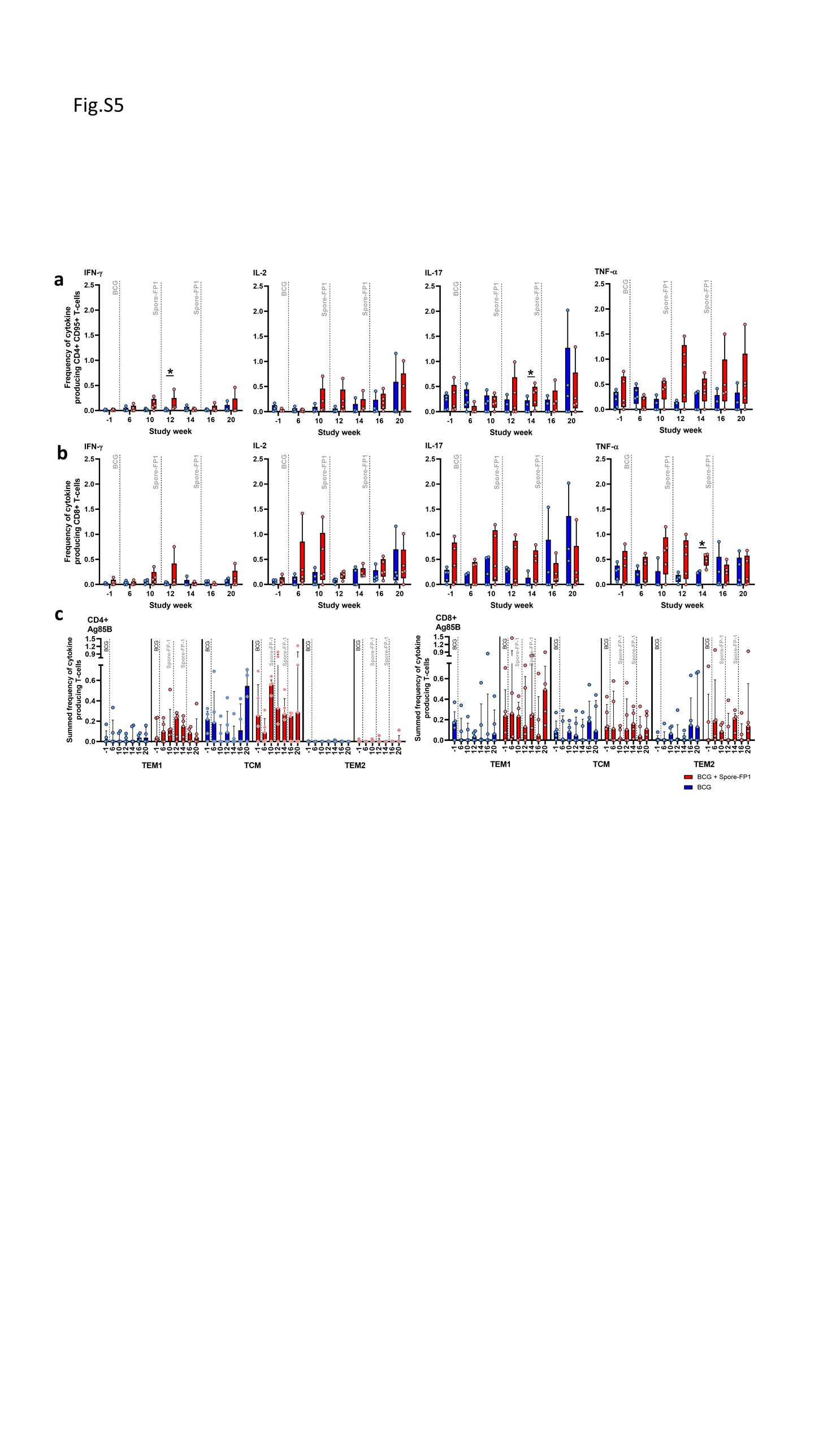

Supplement: Supplementary Figure 5 — Ag85B-specific CD4 and CD8 memory T-cell profiles measured in PBMCs. Plots show the vaccination group median frequency of CD4 T-cells (A) and CD8 T-cells (B) producing the cytokines IFN-γ, IL-2, IL-17 or TNF-α.c) Summed Ag85B-specific CD4 and CD8 memory T-cell subset profiles. Responses were measured prior to (week -1) and following (weeks 6, 10, 12, 14, 16 and 20) BCG vaccination. Bars show the group medians +/− IQR with minimum and maximum values indicated by box whiskers with the dots representing the summed frequency. Significant differences between groups (Mann–Whitney U test) are indicated by bars and asterisks: *P ≤ 0.05 (all P values are unadjusted for multiple comparisons). [file Image_5.tif]

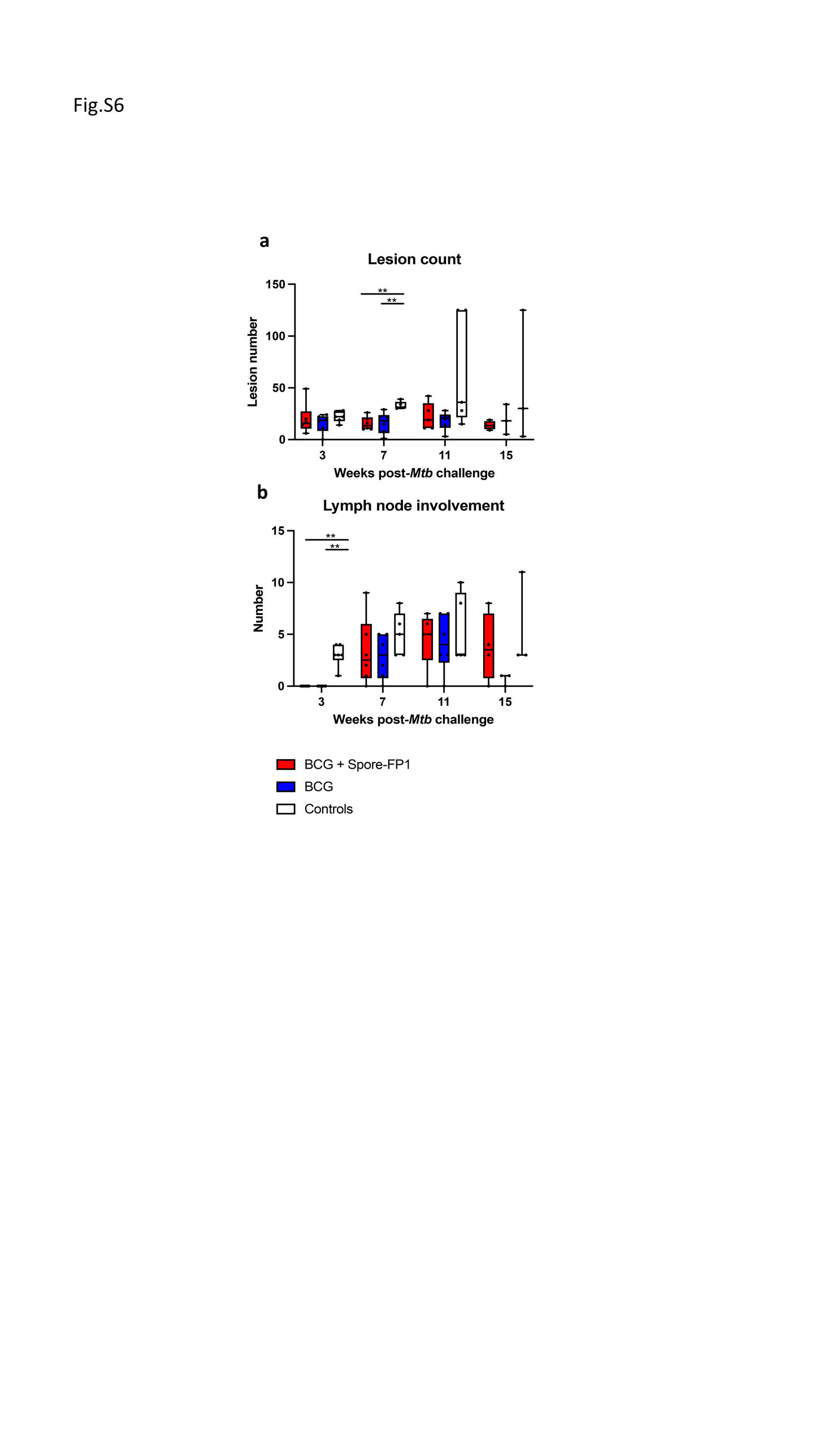

Supplement: Supplementary Figure 6 — Lesion counts (A) and occurrence of lymph node involvement (B) following low dose aerosol Mtb challenge. Box plots show group median (+/- interquartile range) with minimum and maximum values connected by whiskers, unadjusted Mann-Whitney U-test comparison results are reported as: *P ≤ 0.05; **P ≤ 0.01; ***P ≤ 0.001. [file Image_6.tif]

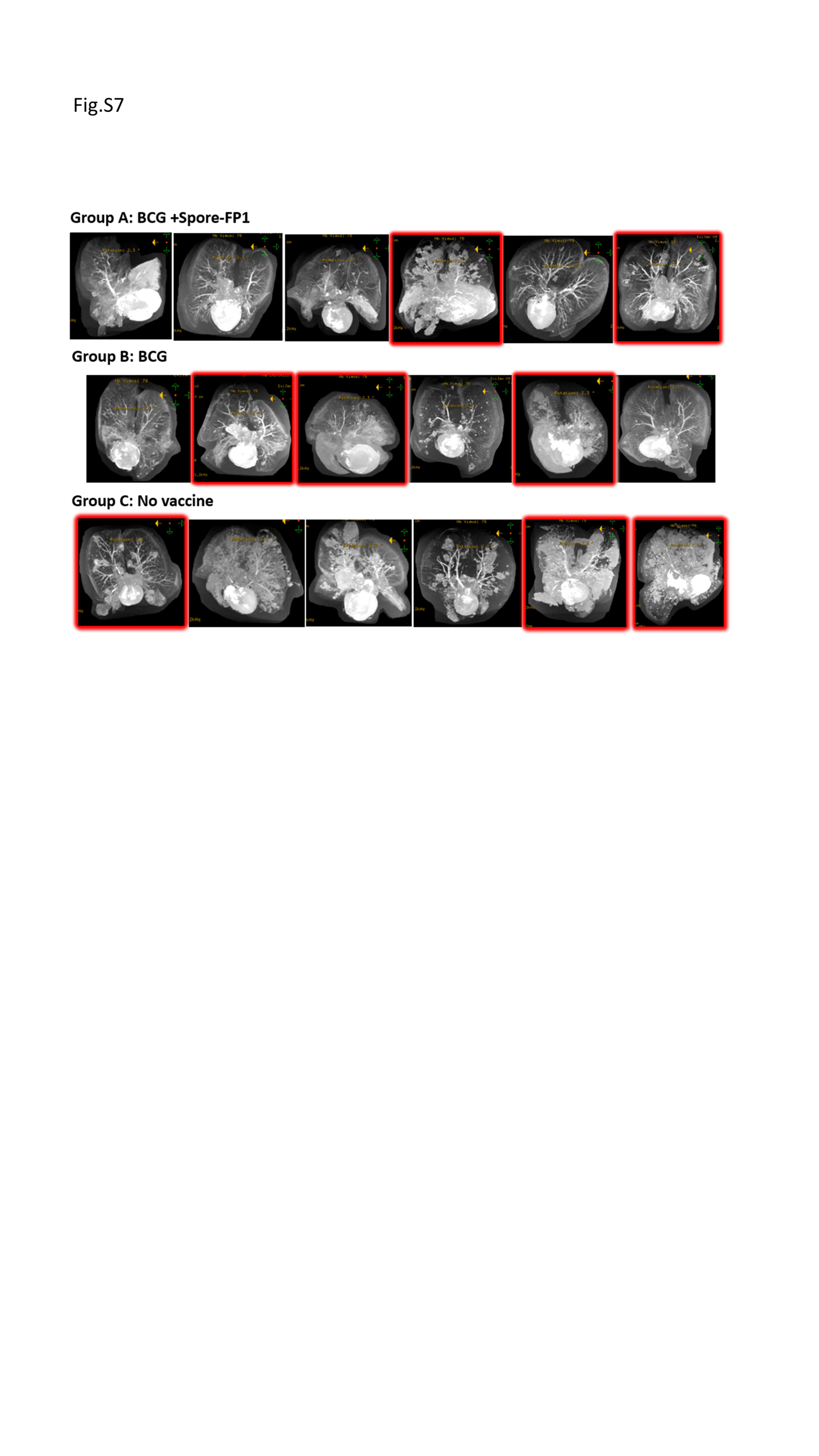

Supplement: Supplementary Figure 7 — Ex-vivo pulmonary MRI scans. Red outlines indicate animals in which disease progressed to meet humane end-point criteria. [file Image_7.tif]

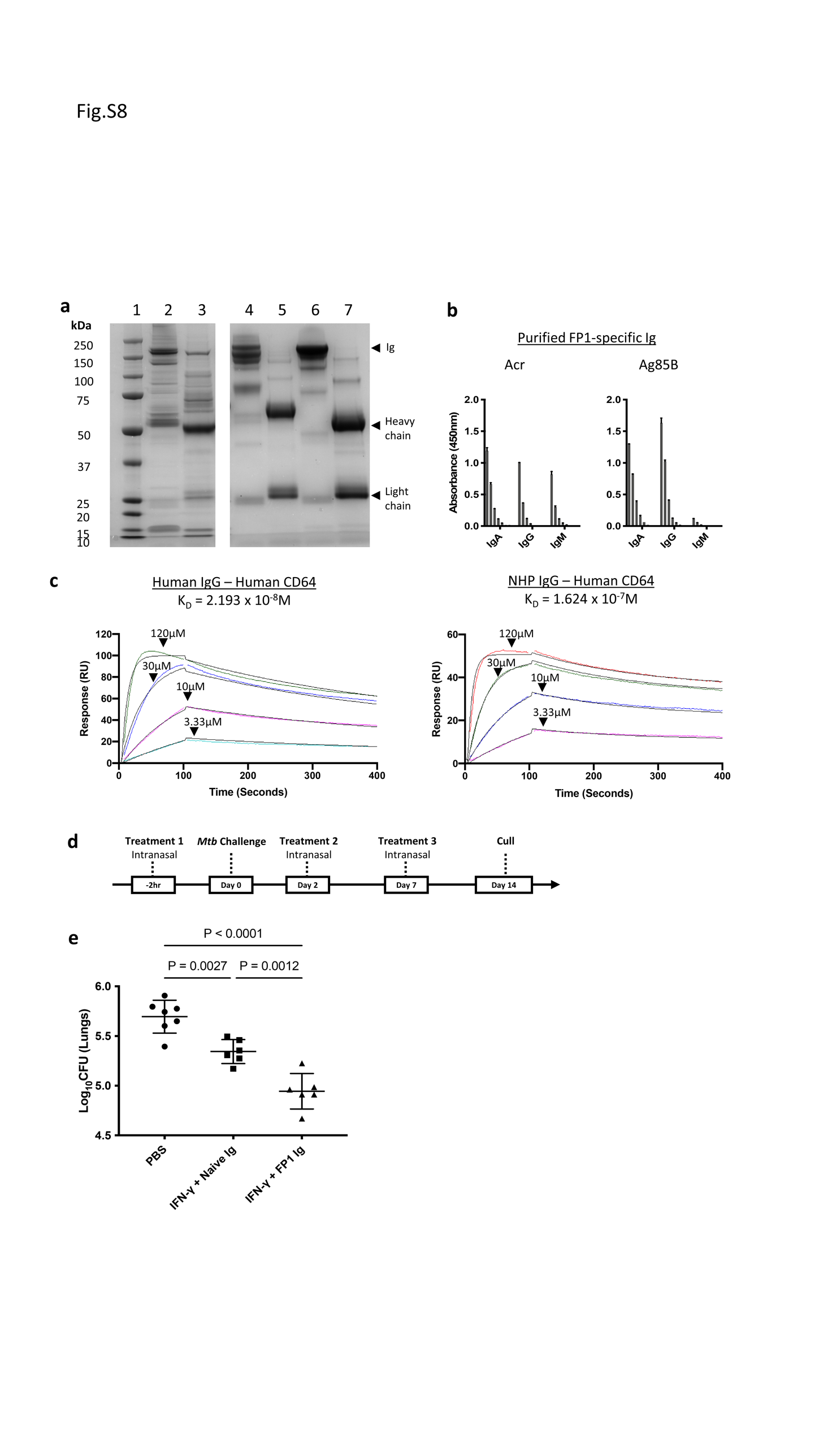

Supplement: Supplementary Figure 8 — Purification of FP1-specific antibodies from immunized NHP and passive transfer to mice; (A) SDS-PAGE analysis of FP1-specific antibodies under reducing (R) or non-reducing (NR) conditions, in comparison to protein G purified fraction of nonimmune human serum; lanes: 1. Standards, 2. NHP Ig (NR), 3. NHP Ig (R), 4. Human serum IgA (NR), 5. Human serum IgA (R), 6. Human IgG (NR), 7. Human IgG (NR). (B) isotype analysis of purified antigen-specific Abs by ELISA (5μg/ml, with 3-fold serial dilutions); (C) Biacore measurement of binding of NHP and human IgG to human high affinity IgG receptor (CD64); shown are concentration dependent response curves, from which an average affinity constant could be deduced for each of the two species. (D) Schematic showing passive transfer of Spore-FP1 immune or BCG-immune NHP antibodies to CD64 Tg mice, followed by Mtb challenge; (E) Lung CFUs from control (untreated, n=7) or treated mice, receiving either BCG-immune NHP IgG + IFN-γ (n=6), or immune (Spore-FP1) antibodies + IFN-γ (n=6). Statistical analysis: One-way ANOVA with Tukey’s post-test. [file Image_8.tif]
